# Can we walk away from cardiovascular disease risk or do we have to ‘huff and puff’? A cross-sectional compositional accelerometer data analysis among adults and older adults in the Copenhagen City Heart Study

**DOI:** 10.1186/s12966-020-00985-w

**Published:** 2020-07-06

**Authors:** Melker Staffan Johansson, Karen Søgaard, Eva Prescott, Jacob Louis Marott, Peter Schnohr, Andreas Holtermann, Mette Korshøj

**Affiliations:** 1 Musculoskeletal Disorders and Physical Workload, National Research Centre for the Working Environment, Lersø Parkallé 105, 2100 Copenhagen Ø, Denmark; 2 Department of Sports Science and Clinical Biomechanics, University of Southern Denmark, Campusvej 55, 5230 Odense M, Denmark; 3 The Copenhagen City Heart Study, Bispebjerg and Frederiksberg Hospital, Nordre Fasanvej 57, Hovedvejen indg. 5, st., 2000 Frederiksberg, Denmark; 4 Department of Cardiology, Bispebjerg University Hospital, Bispebjerg Bakke 23, 2400 Copenhagen NV, Denmark

**Keywords:** Physical activity, Sedentary behaviour, Walking, High-intensity physical activity, Systolic blood pressure, Waist circumference, Low-density lipoprotein cholesterol

## Abstract

**Background:**

It is unclear whether walking can decrease cardiovascular disease (CVD) risk or if high intensity physical activity (HIPA) is needed, and whether the association is modified by age. We investigated how sedentary behaviour, walking, and HIPA, were associated with systolic blood pressure (SBP), waist circumference (WC), and low-density lipoprotein cholesterol (LDL-C) among adults and older adults in a general population sample using compositional data analysis. Specifically, the measure of association was quantified by reallocating time between sedentary behaviour and 1) walking, and 2) HIPA.

**Methods:**

Cross-sectional data from the fifth examination of the Copenhagen City Heart Study was used. Using the software Acti4, we estimated daily time spent in physical behaviours from accelerometer data worn 24 h/day for 7 days (i.e., right frontal thigh and iliac crest; median wear time: 6 days, 23.8 h/day). SBP, WC, and LDL-C were measured during a physical examination. Inclusion criteria were ≥ 5 days with ≥16 h of accelerometer recordings per day, and no use of antihypertensives, diuretics or cholesterol lowering medicine. The 24-h physical behaviour composition consisted of sedentary behaviour, standing, moving, walking, HIPA (i.e., sum of climbing stairs, running, cycling, and rowing), and time in bed. We used fitted values from linear regression models to predict the difference in outcome given the investigated time reallocations relative to the group-specific mean composition.

**Results:**

Among 1053 eligible participants, we found an interaction between the physical behaviour composition and age. Age-stratified analyses (i.e., </≥65 years; 773 adults, 280 older adults) indicated that less sedentary behaviour and more walking was associated with lower SBP among older adults only. For less sedentary behaviour and more HIPA, the results *i)* indicated an association with a lower SBP irrespective of age, *ii)* showed an association with a smaller WC among adults, and *iii)* showed an association with a lower LDL-C in both age groups.

**Conclusions:**

Less sedentary behaviour and more walking seems to be associated with lower CVD risk among older adults, while HIPA types are associated with lower risk among adults. Therefore, to reduce CVD risk, the modifying effect of age should be considered in future physical activity-promoting initiatives.

## Background

Almost 30% of all deaths globally are caused by cardiovascular disease (CVD) [1]. Leading risk factors are high systolic blood pressure (SBP), high waist circumference (WC), and high low-density lipoprotein cholesterol (LDL-C) [2, 3]. Low physical activity levels and excessive sedentary behaviour are associated with these risk factors and incident CVD [2, 4, 5]. Therefore, it is essential to increase physical activity to prevent CVD [4–7]. However, there is need for improved knowledge about feasible and effective physical activity types, and how much that is required to achieve a preventive effect.

For several reasons, *walking* is a physical activity type that has a great potential to prevent CVD. Firstly, walking is safe and easy to integrate in everyday life [8]. Secondly, walking has beneficial effects on several CVD risk factors [9, 10], and reduces the risk of all-cause and CVD-specific mortality [11, 12]. Thirdly, walking may also be easier to communicate and implement in the public compared to physical activity of higher intensity [6]. Walking may hence be one of the most evident physical activity types to promote on a population level to prevent CVD. However, because the age-related decline in maximal aerobic capacity (VO_2_max) [13, 14] leads to a higher relative intensity during walking among elderly, the preventive potential of walking may be highest among older individuals [9]. Younger individuals may therefore need to engage in *high-intensity physical activity (HIPA)* types such as cycling and running to achieve a similar relative intensity and accompanying health benefits [11, 15–17].

Most previous studies have investigated health effects of sedentary behaviour, walking, or HIPA types as being *independent* from other physical behaviours [5, 11, 12, 16, 18]. However, because a day has a fixed duration of time, an increased time spent in one behaviour displaces time available for other behaviours. This means that physical behaviours are *co-dependent* and compositional in nature [19–21]. Therefore, the association between walking, and HIPA, and CVD risk factors depends on how much time that is spent in *other* physical behaviours during the day, and *which* of these an increase in walking or HIPA displaces [22]. For example, if a person walks more and spend less time sedentary, it will likely reduce SBP [9, 23]. On the other hand, if the increase in walking displaces time in HIPA, it will, hypothetically, result in higher SBP over time [24]. Applying standard statistical methods to compositional data [5, 11, 12, 16, 18] has both conceptual and statistical limitations [19–21]; but c*ompositional data analysis (CoDA)* provides tools to analyse such data properly [19].

We are not aware of any studies that have investigated the relationship between *walking* and other physical behaviours (e.g., sedentary behaviour, standing, running, and cycling), and risk factors for CVD using CoDA and device-based measurements of physical behaviours. Some previous studies have used device-based measurements of physical behaviours and CoDA [22, 25–27]. However, they have either used stepping or light and moderate-to-vigorous physical activity (LIPA and MVPA) and not walking and other *types* of physical activity, and the findings of these studies, with regards to SBP, WC, and LDL-C, are inconclusive. It is hence unclear whether it is enough to walk more for adults and older adults, or if HIPA is needed to decrease the risk of CVD. Specific knowledge about the relationship between walking (i.e., measured with devices) and risk factors for CVD is needed to improve our knowledge about walking’s potential to improve public health and decrease the risk of CVD in the general population.

The objectives of this study were to investigate how sedentary behaviour, walking, and HIPA, are associated with risk factors for CVD (i.e., SBP, WC, and LDL-C) among adults and older adults in a general population sample using CoDA. Specifically, the measure of association was quantified by reallocating time between 1) sedentary behaviour and walking, and 2) sedentary behaviour and HIPA, among adults and older adults.

## Methods

### Study design and study population

**Fig. 1:**
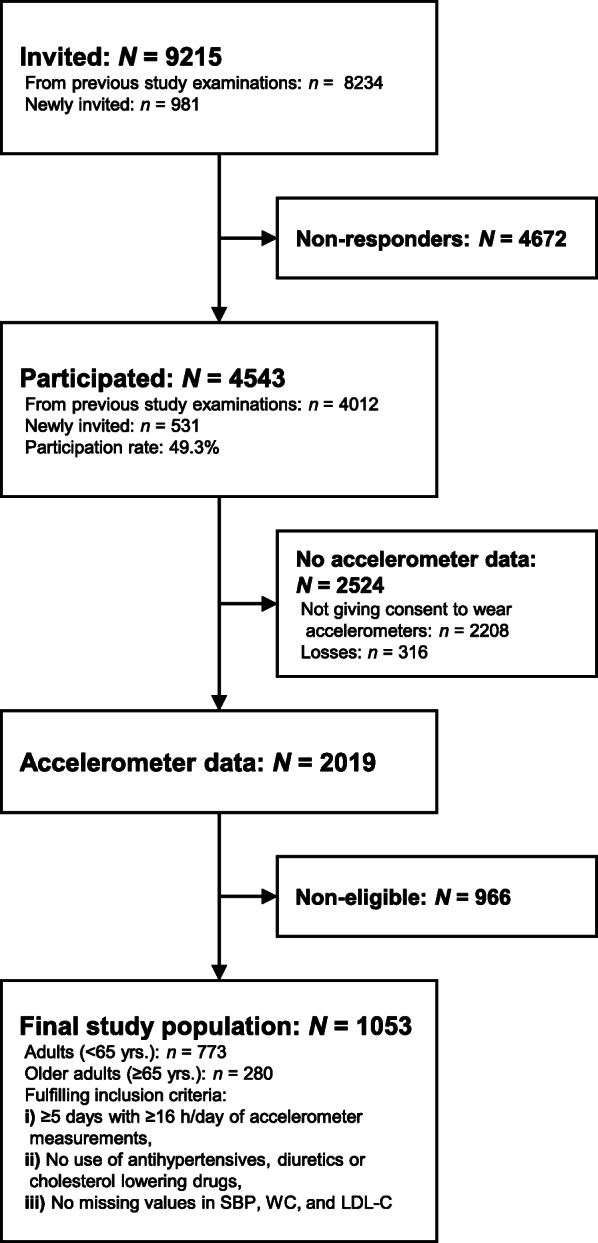
Formation of the final study population of eligible participants in the fifth examination of the Copenhagen City Heart Study. *N*/*n* indicates number of participants

This is a cross-sectional analysis of data collected in the *fifth* examination (October 2011 – February 2015) of the Copenhagen City Heart Study (CCHS) [28]. In total, 9215 individuals were invited of which 4543 participated (49.3%) (Fig. 1). These individuals were ≥20 years old and lived in two parts of Copenhagen, Denmark, and were randomly chosen from the Copenhagen Population Register using a national registration number. Invitations were sent 3 weeks before a scheduled health examination. These included a questionnaire and a pre-paid postcard where the individuals could confirm, decline, or change the appointment. Further details about the source population, recruitment of the initial study population, the invitation procedure, data collection, and data processing are described elsewhere [28, 29].

### Data collection

#### Questionnaire

The study participants filled out a questionnaire covering a wide range of domains including but not limited to socioeconomic status; general, physical and mental health; symptoms and diseases; smoking and alcohol consumption; diet; and medication use. We have made an overview of questions relevant for this study in Additional files, Table A1.

#### Physical examination

All participants were examined at the CCHS’s test centre at a public hospital in the Capital Region of Denmark by trained medical laboratory technicians, medical students and medical specialists.

The tests relevant for this study were measurements of blood pressure, WC, and LDL-C (i.e., our outcomes), and height, and weight (i.e., for descriptive purposes). WC was measured at the approximate midpoint between the lower margin of the last palpable rib and the top of the iliac crest. Three consecutive blood pressure measurements were taken on the participants’ non-dominant arm after 5 min of sitting with an automatic blood pressure monitor (OMRON M3, OMRON Healthcare, Hoofddorp, Netherlands). Venipunctures were taken according to standardised procedures and the level of LDL-C was determined directly (Sanofi Genzyme, Cambridge, Massachusetts, USA). Height was measured without shoes on a fixed scale to the nearest millimetre. Weight was measured with clothes, but without shoes, on a consultation scale (Seca, Hamburg, Germany) to the nearest 100 g.

#### Accelerometer-based measurements of physical behaviours

All participants were invited to participate in a sub-study that involved wearing two tri-axial accelerometers (ActiGraph GT3X+; sampling frequency: 30 Hz; ActiGraph, Pensacola, Florida, USA) 24-h per day for seven consecutive days to measure their daily physical behaviours. In total, 2335 participants gave consent to wear the accelerometers. The accelerometers were attached on **i)** the anterior aspect of the right thigh midway between the greater trochanter and patella oriented along the axis of the thigh, and **ii)** on the lateral aspect of the right iliac crest. They were attached directly to the skin using a double-sided medical tape (Hair-Set for hairpieces; 3 M, Maplewood, Minnesota, USA) and wrapped with transparent adhesive film (OpSite Flexifix; Smith & Nephew, London, UK) to ensure a fixed position during the measurement period.

During the measurement period, the participants were asked to note their leisure time, working hours, time in bed, and periods of non-wear time in a diary. The participants were also asked to only remove the accelerometers in case of adverse skin reactions, discomfort or pain, affected sleep, and when going to a sauna. After the measurement period, the participants returned the accelerometers at the test centre or by mail using a pre-paid envelope. The measurements of physical behaviours have been described in detail in a previous publication [29].

### Processing of raw accelerometer data

#### Detection of physical activity types and stationary behaviours

The MATLAB-software Acti4 (National Research Centre for the Working Environment, Copenhagen, Denmark) was used to detect and derive the time spent in the following physical activity types and stationary behaviours: lying, sitting, standing, moving (i.e., small movements without regular walking while in a standing posture), walking, climbing stairs (i.e., up and down), running, cycling, and rowing. Acti4 detects the physical behaviours through an algorithm that uses inclinations and accelerations [30], with a high sensitivity and specificity [30, 31].

#### Quality control, time in bed and non-wear time

For each individual participant, we visually inspected the activity classification over time to identify and investigate any abnormalities in the data (e.g., high levels of rowing or lack of sitting). *Time in bed* was defined based on a combination of accelerometer and diary data (i.e., bedtime/get up time). *Non-wear time* was ‘operator-defined’ by diary information and visual inspection of the activity classification. In addition, Acti4 detects non-wear time automatically using a set of rules: 1) Periods <10 min without recorded movement were not regarded as non-wear time. 2) Periods between 10 and 90 min were classified as non-wear time if a) the vector sum of the standard deviation of acceleration was >0.5G for any second during a 5-s interval immediate before the period without recorded movement, and b) the accelerometer was placed in a horizontal position (±5°). 3) Periods >90 min without recorded movement were always considered as non-wear time [30]. See previous publication for further details about the processing of the raw accelerometer data [29].

### Eligibility criteria

Our inclusion criteria were: 1) ≥5 days of measurements with ≥16 h of accelerometer recordings per 24-h day, 2) not using antihypertensive, diuretics or cholesterol lowering medicine, and 3) no missing values in any of the outcome variables. All reported ‘sick days’ (i.e., diary information) were excluded.

### Definition of variables

#### Physical behaviour composition

The physical behaviour composition consisted of time (min/24-h day) spent in *sedentary behaviour* (i.e., sum of lying and sitting), *standing*, *moving*, *walking*, *HIPA* (i.e., sum of climbing stairs [up/down], running, cycling, and rowing), and *time in bed*. It hence reflects participants’ 24-h time-use. Time spent in the physical behaviours was accumulated during *waken* hours only (i.e., except for time in bed). Time spent in each behaviour was represented by the individual’s daily mean time across the measurement period standardised to 24 h.

Physical behaviours (i.e., compositional parts) consisting of zeros cannot be included in CoDA. Due to zero time spent climbing stairs, running, cycling, and rowing for some participants, we decided to merge these behaviours into the combined activity class HIPA.

#### Outcomes

We used SBP (mm Hg), WC (cm), and LDL-C (mmol/L) as outcome variables. WC was used rather than BMI or waist-hip ratio since it has been suggested to be a stronger predictor for CVD [3].

#### Covariates and variables for descriptive analyses

In addition to the physical behaviour composition, we used the following covariates in the analyses: sex, age, number of years of education, smoking status, average number of alcohol units/week, and self-reported use of prescribed medication for cardiovascular disease, antidepressants or sedatives, asthma or bronchitis, and diabetes.

For descriptive purposes, body mass index (BMI, calculated as weight in kilograms divided by height in meters squared) was categorised according to the WHO classification into *underweight* (<18.5 kg/m^2^), *normal weight* (18.5- <25.0 kg/m^2^), *overweight* (25.0- <30.0 kg/m^2^), and *obese* (≥30 kg/m^2^) [32]. Furthermore, blood pressure was categorised according to the classification used by the European Society of Hypertension and the European Society of Cardiology into *normal* (systolic: <140 mm Hg and diastolic: <90 mm Hg), *grade 1 hypertension* (systolic: 140- ≤159 mm Hg or diastolic: 90- ≤99 mm Hg), *grade 2 hypertension* (systolic: 160- ≤179 mm Hg or diastolic: 100- ≤109 mm Hg), *grade 3 hypertension* (systolic: ≥180 mm Hg or diastolic: ≥110 mm Hg; i.e., the normal category includes high normal) [33]. Finally, WC was categorised into >88 cm for women and >94 cm for men [3].

An overview including details about how we derived these variables can be found in the Additional files (Table A2).

### Statistical analysis

#### Descriptive statistics

We described the characteristics of the study population using frequencies and percentages (%) or medians and first and third quartiles (Q1-Q3) where appropriate. Medians were used instead of means due to skewed distributions of some of the continuous variables. We described the central tendency and dispersion of the physical behaviour composition with geometric means and a variation matrix, respectively.

#### Investigation of potential selection bias

The characteristics of the non-eligible participants (i.e., having accelerometer data but not fulfilling the eligibility criteria) were compared to the characteristics of the eligible participants. This was done using Mann-Whitney U test, Pearson’s Chi-squared test (i.e., *p*-values <0.05 were considered to indicate differences between groups) and assessing 95% confidence intervals (CI) of medians and proportions. We calculated CIs for medians and proportions using the normal approximation method and the Wilson’s score method, respectively [34].

#### Data transformations

Compositional data is bound to a sample space (i.e., the *simplex*) with a geometry that is incompatible with standard statistical methods. To allow the use of standard statistical methods, we transformed the physical behaviour composition with the *isometric log-ratio* (*ilr*) transformation based on a sequential binary partition process [19]. This resulted in a set of pivot ilr-coordinates that represent the physical behaviour composition in a sample space (i.e., the *real* coordinate space) where standard statistical methods can be applied [20]. Specifically, *pivot ilr*-coordinates were constructed, where the first coordinate (*ilr1*) represents the first part of the composition relative to the geometric mean of the remaining parts [35].

#### Modelling process and time reallocations

We investigated how sedentary behaviour, walking, and HIPA, expressed as ilr-coordinates, were associated with each outcome using linear regression models (i.e., crude and adjusted analyses). Due to the ilr-transformation, the model estimates of the ilr-coordinates are not directly interpretable. A solution to this challenge was to theoretically reallocate time between 1) sedentary behaviour and walking, and 2) sedentary behaviour and HIPA and thereby, quantify the measure of association in an understandable way [20]. This was conducted in the following three steps.

i) For each outcome, we fitted a multiple linear regression model with the ilr-coordinates representing the physical behaviour composition and the previously mentioned covariates (i.e., only in adjusted analyses). The physical behaviour composition as a whole was associated with SBP, WC, and LDL-C in the crude and adjusted analyses (i.e., all *p*-values <0.001, data not shown). We tested for and found an interaction between the physical behaviour composition and age (i.e., p-value for interaction term in the SBP-, WC-, and LDL-C-model: 0.006, <0.001, and <0.001, respectively). Subsequently, all analyses were stratified by age group (i.e., adults <65 years and older adults ≥65 years). We assessed the assumptions of the linear regression models by plotting residuals vs. continuous covariates, residuals vs. fitted values, and by quantile-quantile (Q-Q) plots of the residuals (i.e., assumption of linearity, homogeneous variance of residuals, and assumption of normally distributed residuals). Additionally, we investigated how individuals with extreme ilr-coordinates influenced the model fit by comparing model parameters and the results of the model validation between the ‘full’ models and the models where the few observations with extreme ilr-coordinates had been omitted.
ii) Since the beta-coefficients of the ilr-coordinates are not directly interpretable, we used the reallocation of time between the behaviours to quantify the measure of association in an understandable way. With the age group-specific geometric mean composition as the starting point (i.e., reference composition), we reallocated time according to our study objectives. The time reallocations were made pairwise (i.e., one-to-one). For example, if 10 min were reallocated *from* sedentary behaviour *to* walking in a theoretical reference composition consisting of 580 min sedentary behaviour, 190 min standing, 60 min moving, 90 min walking, 20 min HIPA and 500 min in bed (i.e., 24 h), it would result in **570** min sedentary behaviour and **100** min walking, while all remaining physical behaviours were kept constant.
  For reallocation 1), we reallocated time between sedentary behaviour and walking in 10-min portions. That is, sedentary behaviour was decreased with 10 to 60 min with a corresponding increase in walking time. Similarly, walking was decreased alongside an increase in sedentary time, again from 10 to 60 min. For reallocation 2), we similarly reallocated time between sedentary behaviour and HIPA from 2 to 12 min in 2-min portions.
iii) We estimated the outcome for the reference compositions and each reallocated composition using the fitted values from the regression models. Subsequently, we calculated the *difference* in outcome by subtracting the estimated outcome of the reference composition from the estimated outcome for each reallocated composition [20, 21].

We used the statistical software RStudio (version 1.1.463) [36] running R (version 3.5.3) for all analyses [37]. Specifically, for the analyses involving CoDA, we used the following packages: *compositions* [38] and *robCompositions* [39].

## Results

### Descriptive statistics

The formation of the study population is illustrated in Fig. 1. As previously mentioned, we found an interaction between the physical behaviour composition and age, and have therefore stratified all analyses by age group (i.e., adults <65 years and older adults ≥65 years). We have presented the characteristics of the study population in Table 1. The median accelerometer wear time was 23.8 and 23.9 h/day, and the median number of valid days was 6.0 and 6.0 days among adults and older adults, respectively. The median age was 48.3 and 72.7 years among adults and older adults, respectively. The median SBP, WC, and LDL-C was 127.0 and 143.8 mmHg, 83.0 and 89.0 cm, and 3.0 and 3.3 mmol/L among adults and older adults, respectively.

**Table 1:** Characteristics of 773 adults and 280 older adults of the final study population participating in the fifth examination of the Copenhagen City Heart Study

***N*** = 1053 (100.0%)	Adults (<65 yrs.) ***n*** = 773 (73.4%)	Older adults (≥65 yrs.) ***n*** = 280 (26.6%)
Characteristics	***n*** (%) / Median (Q1-Q3)	***n*** (%) / Median (Q1-Q3)
Accelerometer wear time	773 (100.0)	280 (100.0)
Median minutes/day	1425.00 (1386.29–1440.00)	1432.50 (1390.43–1440.00)
Number of valid days of measurement	773 (100.0)	280 (100.0)
Median number of days	6 (6–7)	6 (6–6)
Sex distribution	773 (100.0)	280 (100.0)
Women	454 (58.73)	154 (55.00)
Men	319 (41.27)	126 (45.00)
Age	773 (100.0)	280 (100.0)
Median years	48.26 (35.29–56.87)	72.70 (68.63–76.00)
Systolic blood pressure	773 (100.0)	280 (100.0)
Median (mm Hg)	127.00 (117.50–137.50)	143.75 (129.38–156.50)
Diastolic blood pressure	773 (100.0)	280 (100.0)
Median (mm Hg)	77.00 (71.00–83.50)	77.50 (70.50–85.00)
Blood pressure classification	773 (100.0)	280 (100.0)
Normal	586 (75.81)	119 (42.50)
Grade 1 hypertension	163 (21.09)	126 (45.00)
Grade 2 or 3 hypertension	24 (3.10)	35 (12.50)
Use of prescribed medication	773 (100.00)	280 (100.00)
Yes	76 (9.83)	49 (17.50)
Waist circumference	773 (100.0)	280 (100.0)
Median (cm)	83.00 (76.00–93.00)	89.00 (81.00–96.25)
Waist circumference (women >80 cm; men >94 cm)	773 (100.0)	280 (100.0)
Under cut-point	195/454 (42.95)	90/154 (58.44)
Over cut-point	97/319 (30.41)	60/126 (47.62)
BMI	773 (100.0)	280 (100.0)
Underweight	10 (1.29)	1 (0.36)
Normal	449 (58.09)	143 (51.07)
Overweight	240 (31.05)	105 (37.50)
Obese	74 (9.57)	31 (11.07)
Low-density lipoprotein cholesterol	773 (100.0)	280 (100.0)
Median (mmol/L)	3.03 (2.47–3.64)	3.34 (2.83–3.90)
Years of education	773 (100.00)	274 (97.86)
Median years	13 (12–14)	10 (8–12)
Level of education	771 (99.74)	278 (99.29)
No further education beyond primary school	71 (9.21)	31 (11.15)
Short education (up to 3 years)	57 (7.39)	37 (13.31)
Vocational or comparable education (1–3 years)	127 (16.47)	83 (29.86)
Higher education (≥3 years)	207 (26.85)	73 (26.26)
University education	309 (40.08)	54 (19.42)
Household income	762 (98.58)	272 (97.14)
Low (<200,000 DKK)	111 (14.57)	79 (29.04)
Middle (200,000–600,000 DKK)	286 (37.53)	153 (56.25)
High (≥600,000 DKK)	365 (47.90)	40 (14.71)
Smoking status	758 (98.06)	274 (97.86)
Current smoker	129 (17.02)	49 (17.88)
Previous smoker	296 (39.05)	134 (48.91)
Non-smoker	333 (43.93)	91 (33.21)
Self-rated fitness compared to peers	771 (99.74)	279 (99.64)
Same	380 (49.29)	127 (45.52)
Better	259 (33.59)	131 (46.95)
Worse	132 (17.12)	21 (7.53)
Self-rated general health	769 (99.48)	276 (98.57)
Excellent or Very good	363 (47.20)	116 (42.03)
Good	304 (39.53)	130 (47.10)
Less good or Poor	102 (13.26)	30 (10.87)

Blood pressure classification is based on the 2013 European Society of Hypertension/European Society of Cardiology guidelines for the management of arterial hypertension: Normal, systolic: <140 mm Hg and diastolic: <90 mm Hg; Grade 1 hypertension, systolic: 140-≤159 mm Hg or diastolic: 90-≤99 mm Hg; Grade 2 hypertension, systolic: 160-≤179 mm Hg or diastolic: 100-≤109 mm Hg; Grade 3 hypertension, systolic: ≥180 mm Hg or diastolic: ≥110 mm Hg (the normal category includes high normal)

BMI was classified according to the WHO classification: Underweight, <18.5 kg/m^2^; Normal weight, 18.5-<25.0 kg/m^2^; Overweight, 25.0-<30.0 kg/m^2^; Obese, ≥30 kg/m^2^

*N/n* number of observations, *y* years, *Q1-Q3* first and third quartile, *BMI* body mass index, *DKK* Danish krone

Of a 24 h-day, adults and older adults spent on average 579.8 and 589.1 min/day in sedentary behaviour, 193.4 and 186.5 min/day standing, 71.1 and 72.9 min/day moving, 85.5 and 74.9 min/day walking, 14.2 and 6.9 min/day in HIPA, and 496.1 and 509.7 min/day in bed, respectively (Table 2). Among both adults and older adults, the highest log-ratio variances were found between HIPA and sedentary behaviour, which reflect a low co-dependency between these behaviours. The lowest log-ratio variances were found between sedentary behaviour and time in bed that reflect a high co-dependency (Table A3 in Additional files).

**Table 2:** Geometric means of 24-h physical behaviour composition among 773 adults and 280 older adults participating in the fifth examination of the Copenhagen City Heart Study

***N*** = 1053	Adults (<65 years) ***n*** = 773	Older adults (≥65 years) ***n*** = 280
Physical Behaviour	Min (%) of a 24-h day	Min (%) of a 24-h day
Sedentary behaviour	579.84 (40.27)	589.07 (40.91)
Standing	193.36 (13.43)	186.49 (12.95)
Moving	71.09 (4.94)	72.92 (5.06)
Walking	85.48 (5.94)	74.90 (5.20)
HIPA	14.16 (0.98)	6.94 (0.48)
Time in bed	496.07 (34.45)	509.68 (35.39)
**Sum**	**1440.00 (100.00**)	**1440.00** (**100.00**)

*HIPA,* high-intensity physical activity which consists of climbing stairs (up/down), running, cycling, and rowing

### Investigation of potential selection bias

The non-eligible study participants had a higher median age, lower level of education, lower household income, a higher proportion used prescribed medication, higher proportions rated their health as *less good* and *poor*, had a higher median SBP (and a higher proportion was classified with hypertension), higher median WC, lower median LDL-C, and higher proportions were classified as overweight and obese compared to those fulfilling the inclusion criteria. See Table A4 in Additional files for details.

### Time reallocations

In both the crude and adjusted analyses, the physical behaviour composition as a whole was associated with SBP, WC, and LDL-C, respectively (i.e., all *p*-values <0.001, data not shown). In the following, we have only included estimates based on the adjusted analyses. For crude estimates, see section *Time reallocations* in Additional files.

#### Systolic blood pressure

Although slightly different in size and precision, both the crude and adjusted estimates of the reallocations suggested the same associations in both age groups (see *Time reallocations*, Additional files).

**Fig. 2:**
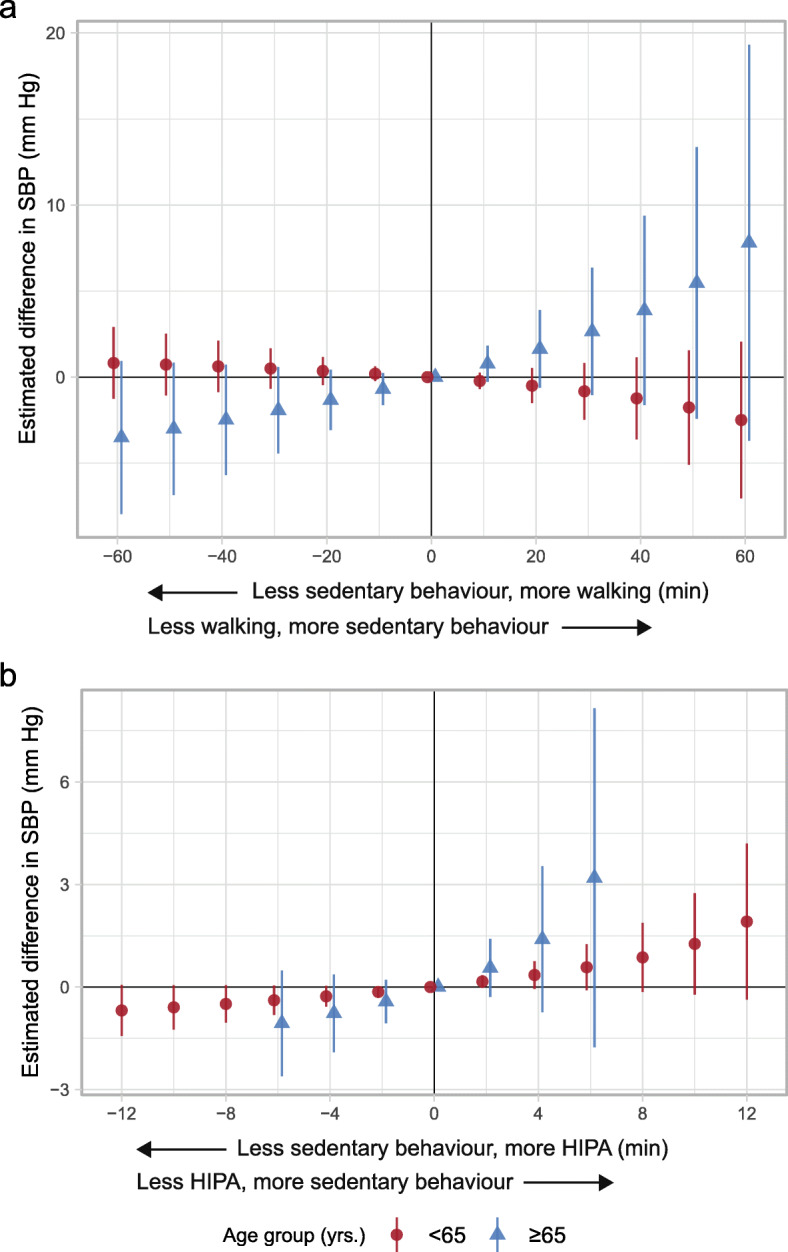
Adjusted estimated difference in systolic blood pressure (SBP, mm Hg, y-axis) given the reallocation of time between sedentary behaviour and **a**) walking, and **b**) HIPA compared to the group-specific reference composition among 773 adults and 280 older adults. A negative value on the x-axis reflects the pairwise reallocation of time *from* sedentary behaviour *to* walking or HIPA, while a positive value reflects the reallocation of time *from* walking or HIPA *to* sedentary behaviour. The origin represent the reference composition (i.e., group-specific geometric mean) that corresponds to i) 579.8 min sedentary behaviour, 193.4 min standing, 71.1 min moving, 85.5 min walking, 14.2 min HIPA, and 496.1 min in bed among adults, and ii) 589.1 min sedentary behaviour, 186.5 min standing, 72.9 min moving, 74.9 min walking, 6.9 min HIPA, and 509.7 min in bed among older adults. The difference in outcome was calculated by subtracting the estimated outcome of the reference composition from the estimated outcome for each reallocated composition. Vertical lines correspond to the 95% confidence intervals. HIPA is high-intensity physical activity (i.e., sum of climbing stairs, running, cycling, and rowing)

Less sedentary behaviour and more walking compared to the reference composition was not associated with an estimated difference in SBP among adults. However, among older adults, the results indicated an associated with a lower SBP (e.g., 30 min: -1.92; 95% CI: -4.43, 0.58 mm Hg) (Fig. 2a and Table 3). In addition, the results indicated less sedentary behaviour and more HIPA relative to the reference composition to be associated with a lower SBP in both age groups (e.g., 6 min among adults: -0.39; 95% CI: -0.82, 0.04 mm Hg; 6 min among older adults: -1.06; 95% CI: -2.61, 0.49 mm Hg) (Fig. 2b and Table 4).

**Table 3:** Estimated adjusted differences in systolic blood pressure, waist circumference, and low-density lipoprotein cholesterol given time reallocations between sedentary behaviour and walking among 773 adults and 280 older adults in the fifth examination of the Copenhagen City Heart Study

Time reallocations (min)	Adults (<65 years) Estimated difference in outcome (95% CI)	Older adults (≥65 years) Estimated difference in outcome (95% CI)
**Systolic blood pressure (mm Hg)**		
-60 min (sedentary behaviour → walking)	0.82 (−1.26, 2.90)	−3.51 (−7.97, 0.94)
-50	0.73 (−1.07, 2.52)	−3.01 (−6.85, 0.84)
-40	0.62 (−0.87, 2.11)	−2.48 (−5.68, 0.72)
-30	0.50 (−0.67, 1.67)	−1.92 (−4.43, 0.58)
-20	0.36 (−0.45, 1.17)	−1.33 (−3.09, 0.42)
-10	0.19 (−0.23, 0.62)	−0.69 (−1.62, 0.23)
0 (reference composition)	0 (0, 0)	0 (0, 0)
10	−0.23 (−0.70, 0.25)	0.77 (−0.28, 1.82)
20	−0.50 (−1.51, 0.52)	1.64 (−0.62, 3.90)
30	−0.83 (−2.47, 0.82)	2.65 (−1.05, 6.35)
40	−1.24 (−3.62, 1.15)	3.88 (−1.62, 9.38)
50	−1.76 (−5.08, 1.55)	5.47 (−2.42, 13.36)
60 min (walking → sedentary behaviour)	−2.49 (−7.05, 2.06)	7.82 (−3.69, 19.32)
**Waist circumference (cm)**		
-60 min (sedentary behaviour → walking)	1.03 (−0.32, 2.38)	−0.41 (−2.63, 1.80)
-50	0.90 (−0.26, 2.06)	−0.32 (−2.24, 1.59)
-40	0.76 (−0.21, 1.72)	−0.24 (−1.83, 1.36)
-30	0.60 (−0.15, 1.35)	−0.16 (−1.41, 1.09)
-20	0.42 (−0.10, 0.95)	−0.10 (−0.97, 0.78)
-10	0.23 (−0.05, 0.50)	−0.04 (−0.50, 0.42)
0 (reference composition)	0 (0, 0)	0 (0, 0)
10	−0.26 (−0.57, 0.05)	0.03 (−0.49, 0.55)
20	−0.56 (−1.21, 0.10)	0.03 (−1.09, 1.16)
30	−0.91 (−1.98, 0.15)	0.01 (−1.83, 1.86)
40	−1.35 (−2.89, 0.20)	−0.05 (−2.78, 2.69)
50	−1.90 (−4.05, 0.25)	−0.17 (−4.10, 3.76)
60 min (walking → sedentary behaviour)	−2.65 (−5.59, 0.30)	−0.43 (−6.15, 5.30)
**Low-density lipoprotein cholesterol (mmol/L)**		
-60 min (sedentary behaviour → walking)	**0.14 (0.02, 0.26)**	0.09 (−0.11, 0.28)
-50	**0.12 (0.02, 0.23)**	0.07 (−0.09, 0.24)
-40	**0.10 (0.01, 0.19)**	0.06 (−0.08, 0.20)
-30	**0.08 (0.01, 0.15)**	0.05 (−0.06, 0.16)
-20	**0.06 (0.01, 0.10)**	0.03 (−0.04, 0.11)
-10	**0.03 (0.004, 0.05)**	0.02 (−0.02, 0.06)
0 (reference composition)	0 (0, 0)	0 (0, 0)
10	**−0.03 (−0.06, −0.01)**	−0.02 (−0.07, 0.02)
20	**−0.07 (−0.13, −0.01)**	−0.05 (−0.14, 0.05)
30	**−0.12 (−0.21, −0.02)**	−0.08 (−0.24, 0.09)
40	**−0.17 (−0.31, −0.03)**	−0.11 (−0.35, 0.13)
50	**−0.23 (−0.43, −0.04)**	−0.16 (−0.50, 0.18)
60 min (walking → sedentary behaviour)	**−0.32 (−0.59, −0.06)**	−0.24 (−0.74, 0.26)

Model adjusted for age, sex, level of education, number of alcohol units/week, smoking status, and use of prescribed medication

Due to missing values in some covariates, 682 and 231 adults and older adults, respectively, were included in the analyses

Reference composition corresponds to i) 579.8 min sedentary behaviour, 193.4 min standing, 71.1 min moving, 85.5 min walking, 14.2 min HIPA, and 496.1 min in bed among adults, and ii) 589.1 min sedentary behaviour, 186.5 min standing, 72.9 min moving, 74.9 min walking, 6.9 min HIPA, and 509.7 min in bed among older adults (i.e., geometric mean)

Estimates in bold indicate 95% CIs not including 0

*CI* confidence interval, *mm Hg* mm of mercury, *mmol/L* mmol per litre, *HIPA* high-intensity physical activity which consists of climbing stairs (up/down), running, cycling, and rowing

**Table 4:** Estimated adjusted differences in systolic blood pressure, waist circumference, and low-density lipoprotein cholesterol given time reallocations between sedentary behaviour and high intensity physical activity among 773 adults and 280 older adults in the fifth examination of the Copenhagen City Heart Study

Time reallocations (min)	Adults (<65 years) Estimated difference in outcome (95% CI)	Older adults (≥65 years) Estimated difference in outcome (95% CI)
**Systolic blood pressure (mm Hg)**		
-12 min (sedentary behaviour → HIPA)	−0.69 (−1.43, 0.06)	–
-10	−0.59 (−1.24, 0.06)	–
-8	−0.49 (−1.04, 0.05)	–
-6	−0.39 (−0.82, 0.04)	−1.06 (−2.61, 0.49)
-4	−0.27 (−0.57, 0.03)	−0.77 (−1.90, 0.36)
-2	−0.14 (−0.30, 0.02)	−0.43 (−1.05, 0.21)
0 (reference composition)	0 (0, 0)	0 (0, 0)
2	0.16 (−0.02, 0.35)	0.56 (−0.29, 1.40)
4	0.35 (−0.05, 0.76)	1.40 (−0.74, 3.53)
6	0.58 (−0.09, 1.25)	3.20 (−1.76, 8.16)
8	0.87 (−0.14, 1.88)	–
10	1.26 (−0.22, 2.75)	–
12 min (HIPA → sedentary behaviour)	1.92 (−0.36, 4.20)	–
**Waist circumference (cm)**		
-12 min (sedentary behaviour → HIPA)	**−1.42 (−1.91, −0.94)**	–
-10	**−1.24 (−1.66, −0.82)**	–
-8	**−1.04 (−1.39, −0.68)**	–
-6	**−0.82 (−1.09, −0.54)**	−0.51 (−1.28, 0.27)
-4	**− 0.57 (−0.77, −0.38)**	−0.36 (−0.93, 0.20)
-2	**− 0.30 (−0.41, −0.20)**	−0.20 (−0.51, 0.11)
0 (reference composition)	0 (0, 0)	0 (0, 0)
2	**0.35 (0.23, 0.47)**	0.26 (−0.16, 0.68)
4	**0.76 (0.50, 1.02)**	0.64 (−0.42, 1.70)
6	**1.26 (0.83, 1.69)**	1.44 (−1.03, 3.91)
8	**1.90 (1.25, 2.55)**	–
10	**2.79 (1.83, 3.75)**	–
12 min (HIPA → sedentary behaviour)	**4.27 (2.80, 5.75)**	–
**Low-density lipoprotein cholesterol (mmol/L)**		
-12 min (sedentary behaviour → HIPA)	**−0.07 (−0.11, −0.02)**	–
-10	**−0.06 (−0.10, −0.02)**	–
-8	**−0.05 (−0.08, −0.02)**	–
-6	**−0.04 (−0.06, −0.01)**	**−0.12 (−0.18, −0.05)**
-4	**−0.03 (−0.05, −0.01)**	**−0.08 (−0.13, −0.04)**
-2	**−0.02 (−0.02, −0.01)**	**−0.05 (−0.07, −0.02)**
0 (reference composition)	0 (0, 0)	0 (0, 0)
2	**0.02 (0.01, 0.03)**	**0.06 (0.03, 0.10)**
4	**0.04 (0.01, 0.06)**	**0.16 (0.07, 0.25)**
6	**0.06 (0.02, 0.10)**	**0.37 (0.15, 0.58)**
8	**0.09 (0.03, 0.15)**	–
10	**0.13 (0.05, 0.22)**	–
12 min (HIPA → sedentary behaviour)	**0.21 (0.07, 0.34)**	–

Model adjusted for age, sex, level of education, number of alcohol units/week, smoking status, use of prescribed medication

Due to missing values in some covariates, 682 and 231 adults and older adults, respectively, were included in the analyses

Reference composition corresponds to i) 579.8 min sedentary behaviour, 193.4 min standing, 71.1 min moving, 85.5 min walking, 14.2 min HIPA, and 496.1 min in bed among adults, and ii) 589.1 min sedentary behaviour, 186.5 min standing, 72.9 min moving, 74.9 min walking, 6.9 min HIPA, and 509.7 min in bed among older adults (i.e., geometric mean)

Estimates in bold indicate 95% CIs not including 0

*CI* confidence interval, *mm Hg* mm of mercury, *mmol/L* mmol per litre, *HIPA* high-intensity physical activity which consists of climbing stairs (up/down), running, cycling, and rowing

#### Waist circumference

Comparing the crude and adjusted analyses, both the size and precision of the estimates differed in both reallocations and in both age groups, except for the reallocation of time between sedentary behaviour and HIPA among adults where the same associations were seen (see *Time reallocations*, Additional files).

**Fig. 3:**
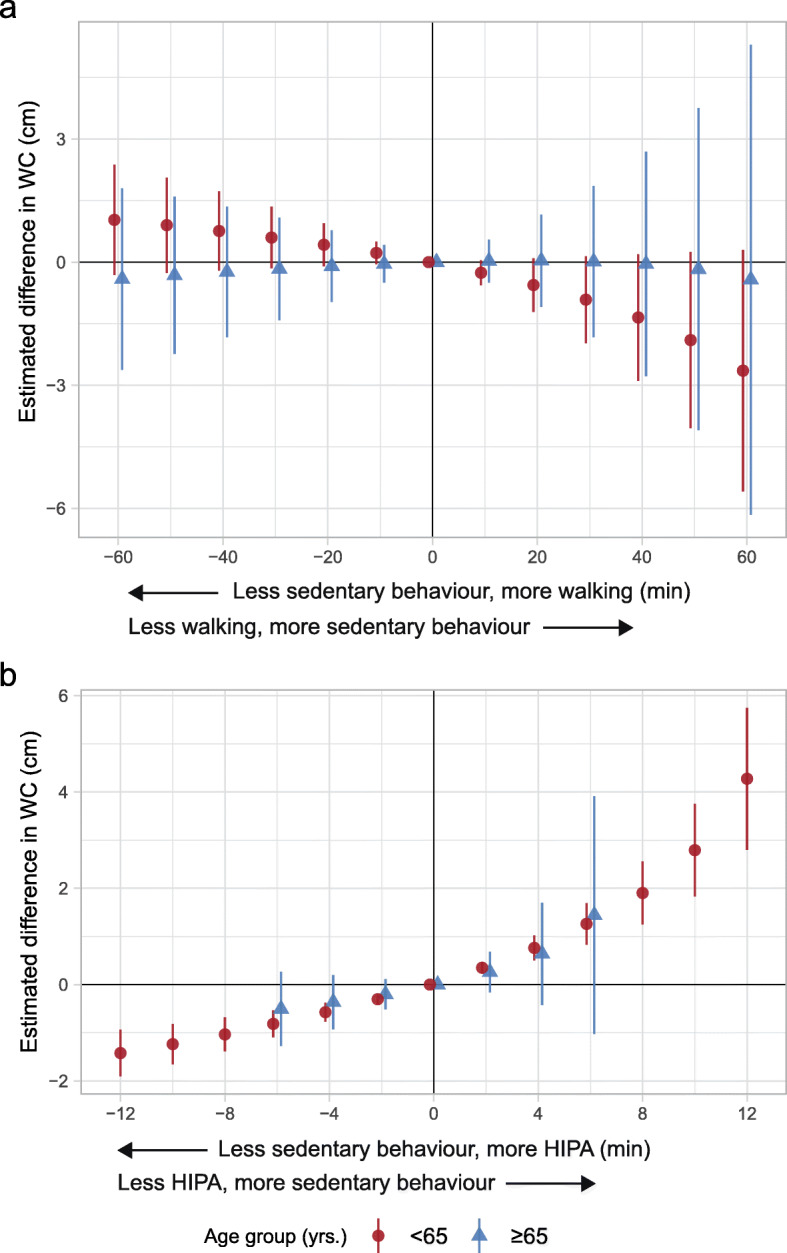
Adjusted estimated difference in waist circumference (WC, cm, y-axis) given the reallocation of time between sedentary behaviour and **a**) walking, and **b**) HIPA compared to the group-specific reference composition among 773 adults and 280 older adults. A negative value on the x-axis reflects the pairwise reallocation of time *from* sedentary behaviour *to* walking or HIPA, while a positive value reflects the reallocation of time *from* walking or HIPA *to* sedentary behaviour. The origin represent the reference composition (i.e., group-specific geometric mean) that corresponds to i) 579.8 min sedentary behaviour, 193.4 min standing, 71.1 min moving, 85.5 min walking, 14.2 min HIPA, and 496.1 min in bed among adults, and ii) 589.1 min sedentary behaviour, 186.5 min standing, 72.9 min moving, 74.9 min walking, 6.9 min HIPA, and 509.7 min in bed among older adults. The difference in outcome was calculated by subtracting the estimated outcome of the reference composition from the estimated outcome for each reallocated composition. Vertical lines correspond to the 95% confidence intervals. HIPA is high-intensity physical activity (i.e., sum of climbing stairs, running, cycling, and rowing)

The results indicated less sedentary behaviour and more walking relative to the reference composition to be associated with a larger WC among adults (e.g., 30 min: 0.60; 95% CI: -0.15, 1.35 cm), while no association was found among older adults (Fig. 3a and Table 3). Furthermore, less sedentary behaviour and more HIPA compared to the reference composition was associated with a smaller WC among adults while only indications of an association were seen among older adults (e.g., 6 min among adults: -0.82; 95% CI: -1.09, -0.54 cm; 6 min among older adults: -0.51; 95% CI: -1.28, 0.27 cm) (Fig. 3b and Table 4).

#### Low-density lipoprotein cholesterol

The crude and adjusted estimates of the time reallocations suggested the same overall association as described in the following (see *Time reallocations*, Additional files).

**Fig. 4:**
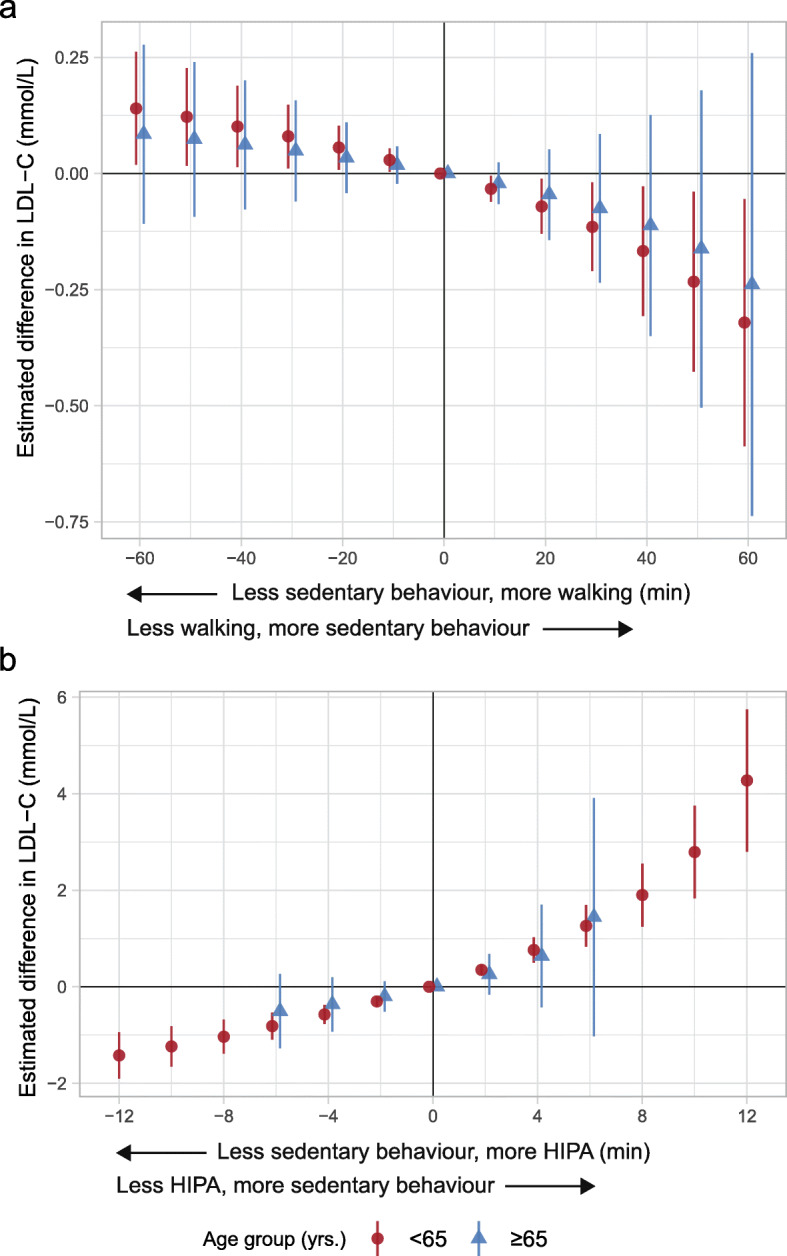
Adjusted estimated difference in low-density lipoprotein cholesterol (LDL-C, mmol/L, y-axis) given the reallocation of time between sedentary behaviour and **a**) walking, and **b**) HIPA compared to the group-specific reference composition among 773 adults and 280 older adults. A negative value on the x-axis reflects the pairwise reallocation of time *from* sedentary behaviour *to* walking or HIPA, while a positive value reflects the reallocation of time *from* walking or HIPA *to* sedentary behaviour. The origin represent the reference composition (i.e., group-specific geometric mean) that corresponds to i) 579.8 min sedentary behaviour, 193.4 min standing, 71.1 min moving, 85.5 min walking, 14.2 min HIPA, and 496.1 min in bed among adults, and ii) 589.1 min sedentary behaviour, 186.5 min standing, 72.9 min moving, 74.9 min walking, 6.9 min HIPA, and 509.7 min in bed among older adults. The difference in outcome was calculated by subtracting the estimated outcome of the reference composition from the estimated outcome for each reallocated composition. Vertical lines correspond to the 95% confidence intervals. HIPA is high-intensity physical activity (i.e., sum of climbing stairs, running, cycling, and rowing)

Among adults, less sedentary and more walking compared to the reference composition was associated with a higher LDL-C (e.g., 30 min: 0.08; 95% CI: 0.01, 0.15 mmol/L). Among older adults, the estimates followed the same pattern but the estimated difference in LDL-C were small and the CIs included zero (Fig. 4a and Table 3). Finally, less sedentary behaviour and more HIPA relative to the reference composition was associated with a lower LDL-C in both age groups (e.g., 6 min among adults: -0.04; 95% CI: -0.06, -0.01 mmol/L; 6 min among older adults: -0.12; 95% CI: -0.18, -0.05 mmol/L) (Fig. 4b and Table 4).

### Model validation

In the SBP- and WC-model among adults, we found slightly curved distributions of the residuals when these were plotted against age (i.e., suggesting non-linearity). The distribution of the residuals was slightly skewed across all outcomes and both age groups, but we chose not to transform any outcome to facilitate understandable results. The variance of the residuals was assessed as homogeneous across all outcomes. See *Linear regression models* in Additional files for details. The few observations with extreme values in some of the *ilr*-coordinates were kept in the analyses since they did not substantially affect the parameters of the models.

## Discussion

### Summary of findings

We found age to modify the association between the physical behaviour composition and SBP, WC, and LDL-C. The results indicated that less sedentary behaviour and more walking compared to the group-specific mean composition was associated with a lower SBP among older adults, but not with the other outcomes in either age group. In addition, the results indicated that less sedentary behaviour and more HIPA was associated with a lower SBP and LDL-C among adults and older adults, and with a smaller WC among adults.

### Interpretation of findings

We found age to modify the association between the physical behaviour composition and our outcomes. This may be most clear in the results for SBP, and indicates that cardiovascular health effects from physical behaviours are age-dependent.

A 2 mm Hg lower SBP has been estimated to lower stroke- and ischaemic heart disease (IHD)-mortality with about 10% and 7%, respectively, on a population level [40, 41]. Our results suggest 60 min less sedentary behaviour and 60 min more walking (compared to the reference composition) to be associated with 3.5 (95% CI: -7.97, 0.94) mm Hg lower SBP among older adults. Among adults, 12 min less sedentary behaviour and 12 min more HIPA was borderline associated with a 0.7 (95% CI: -1.43, 0.06) mm Hg lower SBP. Less sedentary behaviour and more walking among older adults, and more HIPA among all adults, could hence potentially contribute to a lower mean SBP that leads to a shift in the SBP distribution and a decrease in the prevalence of hypertension, which may in turn prevent incident CVD [42]. We emphasise that the CIs in the aforementioned examples include zero; however, and importantly, the majority of the values of the CIs (i.e., 89% and 96%, respectively) indicate a lower SBP given the time reallocations. Therefore, we interpret the results as indicating an association with a lower SBP.

The different effects on SBP among adults and older adults from the time reallocations between sedentary behaviour and *walking* (Fig. 2a) may be explained by several factors. Firstly, one possible explanation to why we did not see any effect among adults may be that physical activity at work and leisure is suggested to have opposite health effects [43, 44] and most of the older adults were not working (i.e., retirement age in Denmark at time of data collection was 65 years). Secondly, as previously discussed, the age-related decrease in VO_2_max [13, 14] results in a higher relative intensity of walking among older adults than adults. This offers another explanation to why walking is suggested to have beneficial effects on SBP among older adults, while adults seem to need more *huffing and puffing* by engaging in HIPA. We acknowledge that ‘overall’ walking may be too heterogeneous from an intensity-perspective to see an effect among adults. However, it is still possible that walking of higher pace could have a beneficial effect. The importance of intensity for SBP is also seen when the estimates are compared across the two time reallocations. For example, among older adults, replacing 4 min of sedentary behaviour with HIPA had a similar effect on SBP to that of replacing 10 min of sedentary behaviour with walking (Fig. 2b).

To our knowledge, there are few previous studies that have used device-based measurements of physical behaviours and a similar analytical approach with which to compare the current results. These previous studies have either used stepping or LIPA and MVPA instead of walking. Since stepping likely includes other types of physical activity than solely walking, and the relative intensity of walking can be either light or moderate (or even vigorous) depending on walking pace [45, 46], it is difficult to compare our results with these studies. However, the current findings related to the reallocation of time from sedentary behaviour to walking among older adults, and the reallocation of time from sedentary behaviour to HIPA in both age groups (i.e., indicating a lower SBP) are incongruent with two previous studies [22, 25], but in agreement with two other studies [26, 27]. It should, however, be emphasised that the two latter studies were conducted in a workplace-setting, and the precision of the estimates is unclear since no CIs were reported.

We found less sedentary behaviour and more HIPA to be associated with a smaller WC in both age groups (e.g., 6 min among adults: -0.8; 95% CI: -1.1, -0.5 cm). It is unclear whether these findings potentially could decrease the CVD risk on a population-level, since the few studies that have investigated the relationship between a change in WC and CVD outcomes have inconclusive findings [47, 48]. Additionally, the estimated differences were smaller than previously reported technical measurement errors of WC [49]. However, even a small shift in the population median of WC might have health implications, since these may affect the prevalence of individuals at risk of CVD (e.g. above or below existing waist circumference thresholds).

There are a limited number of studies using a similar analytical approach with which to compare the results. The findings of the current study, related to the reallocation of time from sedentary behaviour to walking among adults (i.e., indicating an association with a larger WC), are incongruent with one study that did not find an association between less sedentary behaviour and more walking [50]. On the other hand, the corresponding results among older adults (i.e., no association) in the current study are in agreement with this study [50], which was conducted in a workplace-setting (i.e., no older adults were included) and used iso-temporal substitution modelling. Furthermore, the current findings related to the reallocation of time between sedentary behaviour and HIPA in both age groups agree with three previous studies [25, 27, 50].

We found that 6 min less sedentary behaviour and 6 min more HIPA was associated with a -0.04 (95% CI: -0.06, -0.01) and -0.12 (95% CI: -0.18, -0.05) mmol/L lower LDL-C among adults and older adults, respectively. On a population-level, a 1 mmol/L lower non-high-density lipoprotein cholesterol (HDL-C; i.e., total cholesterol minus HDL-C) has been reported to lower IHD-mortality by 30% [51]. Therefore, even small reductions in LDL-C on a population-level could in combination with improvements in other lifestyle factors (e.g., smoking, alcohol consumption and diet) prevent incident IHD [52, 53].

Similar to the literature for SBP and WC, there are few studies that have used device-based measurements of physical behaviours, and a similar analytical approach with which to compare. However, the findings related to the reallocation of time from sedentary behaviour to HIPA are in agreement with a previous workplace-based prospective study that used CoDA [27]. Further, our results from both reallocations are in disagreement with another study, which also used CoDA [25].

Surprisingly, we found less sedentary behaviour and more walking relative to the reference composition to be associated with a *larger* WC among adults (borderline) and a *higher* LDL-C in both age groups (although the CIs included zero among older adults). We do not know how to explain these puzzling findings. However, among adults, one explanation could be differences in occupation, socioeconomic status and health. Specifically, occupations that involve little sedentary behaviour and much walking (i.e., not requiring a high level of education) are often held by individuals with lower socioeconomic status, which in turn is associated with poor health [54], including higher odds of overweight and dyslipidaemia [55].

### Methodological considerations

We emphasise that the cross-sectional study design should be kept in mind when interpreting our results, and that the estimates should be interpreted as measures of association and not causal effects. The risk of reversed causality should be acknowledged, in particular for WC, since evidence suggest a bi-directional relationship between physical behaviours and measurements of adiposity [56]. Importantly, we also emphasise that these findings should be interpreted from a primary prevention perspective since we excluded those taking antihypertensives, diuretics, and cholesterol lowering medicine.

The CIs of some estimates were wide. We believe that a bigger study sample would result in better precision of the estimates, in particular among older adults, which could assist the interpretation of some of our findings.

The sensitivity and specificity of Acti4 has been found to be above 90% across all activity types and stationary behaviours during standardised and semi-standardised conditions, except for climbing stairs that has a lower sensitivity (sensitivity: 75.4%; specificity: 99.7%) [30, 31]. Since Acti4 has not been validated in older populations, we acknowledge that we do not know whether this may have affected the risk of misclassification.

The algorithm in Acti4 cannot differentiate between ascending and descending stairs or between cycling with a light or vigorous effort. This is a limitation, since some physical activity in the HIPA category could be classified as having moderate intensity according to the 2011 Compendium of Physical Activities (i.e., descending stairs, and cycling for leisure [8.9 km/h]) [57, 58]. However, in most situations, climbing stairs, running, cycling, and rowing (i.e., the physical activity types of HIPA) have a MET value that corresponds to vigorous physical activity (i.e., ≥6 METs) [57, 58].

We emphasise that time in bed was based on a combination of diary information about bedtime and visual inspection of the activity classification over time. Our analyses are adjusted for time spent in bed by including it in the regression models. However, since time in bed is only a proxy and not a valid measure of sleep duration or sleep quality, we did not quantify the measure of association between time in bed and the outcomes. Furthermore, cardiorespiratory fitness could be an important effect modifier. However, we could not investigate this, since we do not have any direct measurement of cardiorespiratory fitness.

As in all epidemiological studies using resting blood pressure, there is a risk that some measurements are affected by white coat hypertension or masked hypertension. Future studies may consider to collect ambulatory blood pressure, since this may be a stronger predictor of incident CVD [59]. We acknowledge that the magnitude of measurement error in WC is unclear [49]. The LDL-C measurements were based on non-fasting blood samples, which is in line with routine clinical practice in several countries including Denmark [60]. The maximal mean change in LDL-C after a habitual meal is reported to be clinically non-significant [53, 60]. Therefore, we do not believe that the non-fasting measurements in this study have affected the precision of the LDL-C measurements to a high degree.

We used the group-specific mean time spent in the physical behaviours as reference in all time reallocations. This is important to consider when interpreting our results, since the effect of increasing walking or HIPA at the cost of sedentary behaviour might differ between those with extreme values in some parts of the composition. For example, the effect of reallocating 15 min of sedentary behaviour to walking may be different for an individual that walks 60 min/day compared to the effect of the same reallocation for an individual that only walks 15 min/day (i.e., a 25% vs. 100% relative change in walking time). The estimated differences presented here may hence be less accurate among individuals with more extreme compositions than among those closer to the mean compositions.

### Perspectives

The burden from physical inactivity-related CVD [2] requires massive efforts to get the least active more active [7]. Our results highlight the potential to prevent CVD among older adults by replacing sedentary behaviour with walking, and among both adults and older adults by replacing sedentary behaviour with HIPA. Since individual behavioural change is challenging [61], a systems-based approach to increase physical activity is increasingly recognised [7, 62]. This can involve multi-disciplinary collaborations between researchers and stakeholders where research is designed and solutions created across different societal ‘systems’ (e.g., environment, policy-making and regulation, work-places, communities, health-care, education, etc.) [62]. For example, policy makers can make active transportation by foot or bike more attractive by improving walkability and bicycle infrastructure [63, 64], which is known to increase physical activity and prevent CVD in the population [65]. Other examples include governmental-led programmes that promotes recreational physical activity, such as Get Scotland Walking [66], national or local non-profit organisations that work for healthy community-based sports environments, such as DGI in Denmark [67], and other community-based initiatives such as Parkrun [68]. These can all play important roles on a national, local, or community-level and should be supported. Furthermore, we believe that physical activity could become a more central part of health-care systems if incentives for this were created. Changes in societal systems such as these may hence influence us all to be less sedentary and walk, cycle, or run more while improving our cardiovascular health.

Furthermore, the results in the current study reflect the fact that durations of physical behaviours are co-dependent and highlight the importance of considering all physical behaviours during a 24-h day. To illustrate differences in results from traditional analyses and CoDA, we investigated the association between sedentary behaviour and SBP among adults by fitting a linear regression model adjusted for time spent walking and HIPA using the same data set. This analysis showed that a 10-min increase in sedentary behaviour was associated with a 0.3 mm Hg higher SBP among adults (data not shown). In comparison, our crude analyses based on CoDA showed that 10 min more sedentary behaviour and 10 min less walking (compared to the reference composition) was not associated with an estimated difference in SBP among adults. On the other hand, 10 min more sedentary behaviour and 10 min less HIPA was associated with a 3.4 mm Hg higher SBP (Additional files). This example highlights that analyses based on CoDA can give more nuanced information about the relationship between physical behaviours and a health outcome. The associations between physical behaviours and health are complex, but this must not keep researchers from pushing the field forward by asking important research questions and applying novel methods.

We suggest that future studies use larger study populations (i.e., including older individuals, for example through harmonisation of data from multiple cohorts) with prospective data and CoDA to investigate the health effects of walking, and further investigate the role of domain (i.e., work and leisure) and walking intensity (e.g., using cadence) for cardiovascular health. Finally, our findings may inform intervention studies that target a decrease in sedentary behaviour and an increase in walking or other physical activity types such as cycling and running among both adults and older adults. In particular, the modifying effect of age is important to take into account when planning interventions or physical activity-promoting initiatives.

## Conclusions

We found age to modify the association between physical behaviours and risk factors for CVD. Our findings indicate that less sedentary behaviour and more walking is associated with a lower risk of CVD among older adults, while activity types of higher intensities seem to be associated with a lower risk among adults. To reduce the risk of CVD, the modifying effect of age should be considered in future physical activity-promoting initiatives.

## Supplementary information

**Additional file 1.** Additional files containing an overview of questions and responses from the questionnaire, overview of derived variables, variation matrix, investigation of potential selection bias, results from linear regression models, and results from time reallocations.

## Data Availability

The data generated and analysed during the current study are not publicly available; however, anybody can apply for the use of data by contacting the steering committee of the CCHS [69].

## Abbreviations

CVD: Cardiovascular disease
SBP: Systolic blood pressure
WC: Waist circumference
LDL-C: Low-density lipoprotein cholesterol
VO_2_max: Maximal aerobic capacity
HIPA: High-intensity physical activity
CoDA: Compositional data analysis
CCHS: Copenhagen City Heart Study
USA: United States of America
UK: United Kingdom
BMI: Body mass index
WHO: World Health Organization
Q1-Q3: First and third quartile
CI: Confidence interval
ilr: Isometric log-ratio
LIPA: Light intensity physical activity
MVPA: Moderate-to-vigorous physical activity
IHD: Ischaemic heart disease
HDL-C: High-density lipoprotein cholesterol
