# Supplementary material for: Can we walk away from cardiovascular disease risk or do we have to ‘huff and puff’? A cross-sectional compositional accelerometer data analysis among adults and older adults in the Copenhagen City Heart Study
Source: Int J Behav Nutr Phys Act. 2020 Jul 6;17:84. doi: 10.1186/s12966-020-00985-w (PMC7336624; doi:10.1186/s12966-020-00985-w)
Supplement: Supplementary file 1 — Additional file 1. Additional files containing an overview of questions and responses from the questionnaire, overview of derived variables, variation matrix, investigation of potential selection bias, results from linear regression models, and results from time reallocations. [file 12966_2020_985_MOESM1_ESM.pdf]

Additional files

CAN WE WALK AWAY FROM CARDIOVASCULAR DISEASE RISK OR DO WE HAVE TO 'HUFF AND PUFF'? A COMPOSITIONAL ACCELEROMETER DATA ANALYSIS AMONG ADULTS AND OLDER ADULTS IN THE COPENHAGEN CITY HEART STUDY

Melker S. Johansson[a, b], Karen Søgaard[b], Eva Prescott[c, d], Jacob L. Marott[c], Peter Schnohr[c], Andreas Holtermann[a] and Mette Korshøj[a]

[a] Musculoskeletal Disorders and Physical Workload, National Research Centre for the Working Environment, Copenhagen, Denmark

[b] Department of Sports Science and Clinical Biomechanics, University of Southern Denmark, Odense, Denmark

[c] The Copenhagen City Heart Study, Bispebjerg and Frederiksberg Hospital, Frederiksberg, Denmark

[d] Department of Cardiology, Bispebjerg University Hospital, Copenhagen, Denmark

Table of contents

1 Overview of questions and responses from questionnaire 3
2 Overview of derived variables 4
3 Variation matrix 7
4 Investigation of potential selection bias 8
5 Linear regression models 10
5.1 Interpretation of the first iso-metric log-ratio coordinate 10
5.2 Systolic blood pressure 10
5.2.1 Crude model 10
5.2.2 Adjusted model 11
5.2.3 Model validation 12
5.3 Waist circumference 13
5.3.1 Crude model 13
5.3.2 Adjusted model 14
5.3.3 Model validation 15
5.4 Low-density lipoprotein cholesterol 16
5.4.1 Crude model 16
5.4.2 Adjusted model 17
5.4.3 Model validation 18
6 Time reallocations 19
6.1 Systolic blood pressure 19
6.1.1 Crude 19
6.1.2 Adjusted 21
6.2 Waist circumference 22
6.2.1 Crude 22
6.2.2 Adjusted 24
6.3 Low-density lipoprotein cholesterol 25
6.3.1 Crude 25
6.3.2 Adjusted 27

1 Overview of questions and responses from questionnaire

Table A1. Overview of questions and responses used in the present study from the questionnaire in the fifth examination of the Copenhagen City Heart Study (Denmark)

Question	Possible responses
Number of years of education	
How many years have you gone to school? (primary or secondary school, high school or similar) (max. 14 years)	Participants filled out the number of years in free text.
Level of education	
What education have you completed since you left municipal primary and lower secondary school?	No education<br>Short education (≤3 years with books)<br>Vocational or similar education (1-3 years)<br>Higher education (≥3 years, e.g., teacher, nurse or similar)<br>University education
Household income	
What was your total household income before tax last year?	<100 000 DKK<br>100 000 – 200 000 DKK<br>200 000 – 400 000 DKK<br>400 000 – 600 000 DKK<br>600 000 – 800 000 DKK<br>>800 000 DKK
Smoking status	
Do you smoke?<br>If no, have you previously smoked?	Yes<br>No
Self-rated fitness compared to peers	
How do you rate your fitness compared to your peers?	Same<br>Better<br>Worse
Self-reported general health	
How do you think your health is all in all?	Excellent<br>Very good<br>Good<br>Less good<br>Poor

All questions have been freely translated from Danish to English for the purpose of this overview only.
DKK, Danish krone

2 Overview of derived variables

**Table A2.** Overview of derived variables used in the present study, based on data from the questionnaire and physical examination in the fifth examination of the Copenhagen City Heart Study (Denmark)	
**Variable and question**	**Possible responses and definition**
Covariates	
Sex	
	Women<br>Men<br>Derived from civil registration number<br>Number of missing values: 0
Age	
	Age at date of physical examination, derived from date of birth.<br>Number of missing values: 0
Number of years of education	
"*How many years have you gone to school? (primary or secondary school, high school or similar) (max. 14 years)*"	Participants filled out the number of years in free text.<br>Number of missing values: 0
Average number of alcohol units per week	
Participants were asked about the average number of beer(s) (33 cl), white wine (12.5 cl), red wine (12.5 cl), liqueur (8 cl) or spirits (4 cl) per week.	Participants filled out the number of units in free text.<br>The average number of units was calculated by taking the sum of the reported alcohol consumption.<br>In case of all values missing, the participant was assigned a missing value.<br>Number of missing values: 79 among adults, 38 among older adults
Smoking status	
Study participants were categorised as smokers, previous smokers and non-smokers based on the questions *"Do you smoke?"* and *"If no, have you previously smoked?"* with response categories *"Yes"* and *"No"*.	Those answering "Yes" to "*Do you smoke?*" were categorised as smokers.<br>Those answering "No" to "*Do you smoke?*", and "*If no, have you previously smoked?*" were categorised as non-smokers.<br>Those answering "Yes" to "*If no, have you previously smoked?*" were categorised as previous smokers.<br>Number of missing values: 15 among adults, 6 among older adults
Self-reported use of prescribed medication	
"Do you daily or almost daily take:"	Participants indicated their answer ("Yes" or "No") for each listed class of medication.<br>Those answering "Yes" to anticoagulants, antihypertensives, other heart medicine, diuretics, cholesterol lowering drugs, antidepressants or sedatives, or insulin or other medication for diabetes were considered to use prescribed medicine.<br>Those answering "No" to the abovementioned items (or leaving items blank) were classified as not taking prescribed medicine.<br>If all items were missing, the participant was assigned a missing value.<br>Number of missing values: 0

**Table A2.** (Continued)	
Variables for descriptive purposes	
BMI	
BMI was derived from measured height and weight (weight [kg] / (height [m])^2 = BMI [kg/m^2])	BMI was categorised into (according to WHO classification):<br>*Underweight* (<18.5 kg/m^2)<br>N*ormal weight* (18.5-<25.0 kg/m^2)<br>*Overweight* (25.0-<30.0 kg/m^2)<br>*Obese* (≥30 kg/m^2<br>Number of missing values: 0
Blood pressure classification	
Blood pressure was measured as part of the physical examination.	Blood pressure was categorised into:<br>*Normal* (systolic: <140 mm Hg and diastolic: <90 mm Hg; i.e., including high normal)<br>*Grade 1 hypertension* (systolic: 140-≤159 mm Hg or diastolic: 90-≤99 mm Hg)<br>*Grade 2 hypertension* (systolic: 160-≤179 mm Hg or diastolic: 100-≤109 mm Hg)<br>*Grade 3 hypertension* (systolic: ≥180 mm Hg or diastolic: ≥110 mm Hg)<br>Number of missing values: 0
Waist circumference (categorised)	
Waist circumference was measured as part of the physical examination.	WC was categorised into:<br>>88 cm for women<br>>94 cm for men.<br>Number of missing values: 0
Household income	
"*What was your total household income before tax last year?*"	Response categories:<br><100 000 DKK<br>100 000 to 200 000 DKK<br>200 000 to 400 000 DKK<br>400 000 to 600 000 DKK<br>600 000 to 800 000 DKK<br>>800 000 DKK<br>Response categories grouped into:<br>Low (<200 000 DKK),<br>Moderate (200 000 to 600 000 DKK)<br>High (>600 000 DKK)<br>Number of missing values: 8 among adults, 11 among older adults
Self-rated fitness compared to peers	
"*How do you rate your fitness compared to your peers?*"	Response categories:<br>Same<br>Better<br>Worse<br>Number of missing values: 2 among adults, 1 among older adults

**Table A2.** (Continued)	
Self-rated general health	
"How do you think your health is all in all?"	Response categories:<br>Excellent<br>Very good<br>Good<br>Less good<br>Poor<br>Response categories grouped into:<br>Excellent and very good<br>Good<br>Less good and poor<br>Number of missing values:
All questions have been freely translated from Danish to English for the purpose of this overview only.<br>BMI, body mass index; WHO's classification: underweight, <18.5 kg/m^2; normal, 18.5-<25.0 kg/m^2; overweight, 25.0-<30.0 kg/m^2; obese, ≥30 kg/m^2. The underweight category was merged with the normal weight category because of low number of underweight individuals.<br>WC, waist circumference<br>DKK, Danish krone	

3 Variation matrix

Table A3. Variation matrix of parts in physical behaviour composition among 773 adults and 280 older adults participating in the fifth examination of the Copenhagen City Heart Study (Denmark)

**Older adults**	**Adults**					
Physical behaviour	*Sedentary*	*Standing*	*Moving*	*Walking*	*HIPA*	*Time in bed*
Sedentary	0	0.260	0.244	0.213	1.094	0.066
Standing	0.306	0	0.110	0.170	1.002	0.145
Moving	0.305	0.126	0	0.108	0.984	0.163
Walking	0.309	0.262	0.213	0	1.019	0.153
HIPA	1.646	1.449	1.358	1.233	0	0.974
Time in bed	0.070	0.215	0.220	0.256	1.570	0

Values in the upper right diagonal corresponds to adults (<65 yrs.) and values in the lower left diagonal are older adults (≥65 yrs.). Small values indicate a high co-dependency between two parts of the physical behaviour composition, and vice versa for large values.
HIPA, high-intensity physical activity which consists of climbing stairs (up/down), running, cycling and rowing
Green indicate values in the range >0 to ≤0.5; orange indicate values in the range >0.5 to ≤1.0; red indicate values >1.0.

4 Investigation of potential selection bias

Table A4. Comparison of characteristics of 966 non-eligible and 1053 eligible participants from the fifth examination of the Copenhagen City Heart Study (Denmark)

***N* = 2019**	**Non-eligible *n* = 966**	**Final study population *n* = 1053**	***P***
**Characteristics**	**n (%) [95% CI] Median [95% CI]**	**n (%) [95% CI] Median [95% CI]**	
Accelerometer wear time			**<0.001**
Median minutes/day	1435.191 [1432.5-1437.858]	1427.5 [1425-1430]	
Number of valid days of measurement			**<0.001**
Median number of days	6 [6-6]	6 [6-6]	
Sex distribution			0.99
Women	559 (57.9%) [54.7-60.9]	608 (57.7%) [54.7-60.7]	
Men	407 (42.1%) [39.1-45.3]	445 (42.3%) [39.3-45.3]	
Age			**<0.001**
Median years	65.749 [64.715-66.872]	54.886 [54.141-55.729]	
Years of education			**<0.001**
Median years	11 [11-11]	12 [12-12]	
Level of education			**<0.001**
No [further] education	121 (12.6%) [10.6-14.8]	102 (9.7%) [8.1-11.7]	
Short education (up to 3 years)	106 (11%) [9.2-13.1]	94 (9%) [7.4-10.8]	
Vocational or comparable education (1-3 years)	290 (30.1%) [27.3-33.1]	210 (20%) [17.7-22.5]	
Higher education (≥3 years)	231 (24%) [21.4-26.8]	280 (26.7%) [24.1-29.5]	
University education	216 (22.4%) [19.9-25.1]	363 (34.6%) [31.8-37.5]	
Household income			**<0.001**
Low (<200 000 DKK)	244 (25.8%) [23.1-28.7]	190 (18.4%) [16.1-20.9]	
Middle (200 000-600 000 DKK)	460 (48.6%) [45.5-51.8]	439 (42.5%) [39.5-45.5]	
High (≥600 000 DKK)	242 (25.6%) [22.9-28.5]	405 (39.2%) [36.2-42.2]	
Smoking status			0.482
Non-smoker	367 (38.5%) [35.5-41.6]	424 (41.1%) [38.1-44.1]	
Previous smoker	419 (44%) [40.8-47.1]	430 (41.7%) [38.7-44.7]	
Current smoker	167 (17.5%) [15.2-20.1]	178 (17.2%) [15.1-19.7]	
Average weekly number of units of alcohol per week			**0.047**
Median units/week	7 [7-8]	7 [6-7]	
Use of prescribed medication			**<0.001**
No	273 (28.5%) [25.7-31.4]	928 (88.1%) [86-89.9]	
Yes	685 (71.5%) [68.6-74.3]	125 (11.9%) [10.1-14]	

**Table A4.** (Continued)			
Self-rated fitness compared to peers			0.413
Same	446 (46.4%) [43.3-49.6]	507 (48.3%) [45.3-51.3]	
Better	355 (36.9%) [33.9-40]	390 (37.1%) [34.3-40.1]	
Worse	160 (16.6%) [14.4-19.1]	153 (14.6%) [12.6-16.8]	
Self-rated general health			**<0.001**
Excellent or Very good	347 (36%) [33.1-39.1]	479 (45.8%) [42.8-48.9]	
Good	415 (43.1%) [40-46.2]	434 (41.5%) [38.6-44.5]	
Less good or Poor	201 (20.9%) [18.4-23.6]	132 (12.6%) [10.8-14.8]	
Systolic blood pressure			**<0.001**
Median (mm Hg)	139 [138-140]	130.5 [129-131]	
Diastolic blood pressure			0.621
Median (mm Hg)	77.5 [77-78]	77 [76.5-78]	
Blood pressure classification			**<0.001**
Normal	480 (50.5%) [47.4-53.7]	705 (67%) [64.1-69.7]	
Grade 1 hypertension	371 (39.1%) [36-42.2]	289 (27.4%) [24.8-30.2]	
Grade 2 or 3 hypertension	99 (10.4%) [8.6-12.5]	59 (5.6%) [4.4-7.2]	
Waist circumference			**<0.001**
Median (cm)	90 [90-91]	85 [84-86]	
WC (women >80 cm, men >94 cm)			**<0.001**
Under cut-point	374 (39%) [36-42.2]	611 (58%) [55-61]	
Above cut-point	584 (61%) [57.8-64]	442 (42%) [39-45]	
BMI, WHO classification			**<0.001**
Normal (incl. underweight)	386 (40.1%) [37-43.2]	603 (57.3%) [54.3-60.2]	
Overweight	405 (42.1%) [39-45.2]	345 (32.8%) [30-35.7]	
Obese	172 (17.9%) [15.6-20.4]	105 (10%) [8.3-11.9]	
Low-density lipoprotein cholesterol			**<0.001**
Median (mmol/L)	2.84 [2.76-2.89]	3.11 [3.04-3.16]	

The non-eligible includes 191 individuals with missing information on whether they agreed to wear accelerometers or not.
N/n, number of observations
P, p-value from Mann-Whitney U test and Pearson's Chi-squared test
CI, confidence interval, CIs were calculated using the Wilson score method for proportions and the normal approximation method for medians.
DKK, Danish krone
Blood pressure classification is based on the 2013 European Society of Hypertension/European Society of Cardiology guidelines for the management of arterial hypertension (the normal category includes high normal).
WC, waist circumference
BMI, body mass index; WHO's classification: underweight, <18.5 kg/m^2; normal, 18.5-<25.0 kg/m^2; overweight, 25.0-<30.0 kg/m^2; obese, ≥30 kg/m^2. The underweight category was merged with the normal weight category because of low number of underweight individuals. The normal BMI-category includes 9 and 11 underweight individuals in the non-eligible and eligible participants, respectively.

5 Linear regression models

5.1 Interpretation of the first iso-metric log-ratio coordinate

The model estimates of the iso-metric log-ratio (ilr) coordinates are not directly interpretable due to the ilr-transformation. The first coordinate (ilr1) represent sedentary behaviour relative to the geometric mean of the remaining behaviours in the daily physical behaviour composition. The direction of the association between sedentary behaviour relative to the remaining physical behaviours and the outcome is therefore reflected by the sign of the model estimate of the ilr1-coordinate. For example, in Table A5.1.1.1., the estimate of ilr1 is positive (i.e., 9.57) and the 95% confidence interval does not include 0. This means that a one-unit increase in time spent in sedentary behaviour, with a corresponding decrease in the remaining physical behaviours, is associated with a higher systolic blood pressure. Importantly, the unit of ilr1 is not interpretable, and it is unclear whether a decrease in one of the remaining physical behaviours are driving this association more than other behaviours. Therefore, the measure of association was quantified by reallocating time between the physical behaviours of interest (see section 6 Time reallocations).

5.2 Systolic blood pressure

5.2.1 Crude model

5.2.1.1 Adults

Table A5.1.1.1. Model output from crude systolic blood pressure model among 773 adults

Variable	Estimate	Standard error	95% CI	
			Lower	Upper
Intercept	119.8632	3.558893	112.8768	126.8495
ilr1	9.576964	2.67568	4.324438	14.82949
ilr2	-0.25579	2.578211	-5.31698	4.805395
ilr3	3.174703	2.54205	-1.8155	8.164903
ilr4	3.988626	2.263486	-0.45474	8.431987
ilr5	2.272421	2.440449	-2.51833	7.063174

CI, confidence interval
ilr1-ilr5, iso-metric log-ratio coordinates representing the transformed physical behaviour composition
ilr1 represent sedentary behaviour relative to the geometric mean of the remaining behaviours in the daily physical behaviour composition

5.2.1.2 Older adults

Table A5.1.1.2. Model output from crude systolic blood pressure-model among 280 older adults

Variable	Estimate	Standard error	95% CI	
			Lower	Upper
Intercept	122.6674	6.166479	110.5277	134.8071
ilr1	9.768649	5.075413	-0.22311	19.76041
ilr2	8.999392	4.382527	0.371688	17.6271
ilr3	4.599849	4.35513	-3.97392	13.17362
ilr4	-3.12228	3.729011	-10.4634	4.218876
ilr5	1.148204	4.002177	-6.73072	9.027128

CI, confidence interval
ilr1-ilr5, iso-metric log-ratio coordinates representing the transformed physical behaviour composition
ilr1 represent sedentary behaviour relative to the geometric mean of the remaining behaviours in the daily physical behaviour composition

5.2.2 Adjusted model

5.2.2.1 Adults

**Table A5.1.2.1.** Model output from adjusted systolic blood pressure-model among 682 adults				
**Variable**	**Estimate**	**Standard error**	**95% CI**	
			**Lower**	**Upper**
Intercept	86.16183	7.470494	71.4934	100.8303
ilr1	4.567675	2.61704	-0.57093	9.706275
ilr2	0.159965	2.439412	-4.62986	4.949789
ilr3	-5.17022	2.445392	-9.97179	-0.36865
ilr4	2.17503	2.184782	-2.11482	6.464885
ilr5	-1.00135	2.38596	-5.68622	3.683517
Age	0.504823	0.052673	0.401399	0.608247
Sex<br>Men vs. Women	9.288953	1.230714	6.872425	11.70548
Level of education	0.249837	0.40943	-0.55409	1.053759
Smoking status<br>Previous smoker vs. Never smoker	1.382264	1.262018	-1.09573	3.860257
Smoking status<br>Smoker vs. Never smoker	4.01337	1.717305	0.641415	7.385326
Average units of alcohol/week	0.115185	0.068137	-0.0186	0.248972
Use of prescribed medicine<br>Yes vs. No	-1.30652	1.950499	-5.13635	2.523321

CI, confidence interval
ilr1-ilr5, iso-metric log-ratio coordinates representing the transformed physical behaviour composition
ilr1 represent sedentary behaviour relative to the geometric mean of the remaining behaviours in the daily physical behaviour composition

5.2.2.2 Older adults

**Table A5.1.2.2.** Model output from adjusted systolic blood pressure-model among 231 older adults				
**Variable**	**Estimate**	**Standard error**	**95% CI**	
			**Lower**	**Upper**
Intercept	112.7153	19.05417	75.1613	150.2693
ilr1	10.04899	5.709754	-1.20439	21.30238
ilr2	7.038248	4.831675	-2.48453	16.56102
ilr3	1.726912	5.087563	-8.3002	11.75402
ilr4	-0.46666	4.234231	-8.81193	7.878611
ilr5	3.056655	4.875565	-6.55262	12.66593
Age	0.394619	0.25287	-0.10376	0.893001
Sex<br>Men vs. Women	0.530119	2.796754	-4.98202	6.042257
Level of education	-1.15681	0.594629	-2.32877	0.01515
Smoking status<br>Previous smoker vs. Never smoker	-0.5132	3.020691	-6.46669	5.440301
Smoking status<br>Smoker vs. Never smoker	4.993801	4.183403	-3.25129	13.23889
Average units of alcohol/week	-0.02352	0.165186	-0.34909	0.302045
Use of prescribed medicine<br>Yes vs. No	-2.94745	3.666266	-10.1733	4.278414

CI, confidence interval
ilr1-ilr5, iso-metric log-ratio coordinates representing the transformed physical behaviour composition
ilr1 represent sedentary behaviour relative to the geometric mean of the remaining behaviours in the daily physical behaviour composition

5.2.3 Model validation

5.2.3.1 Adults

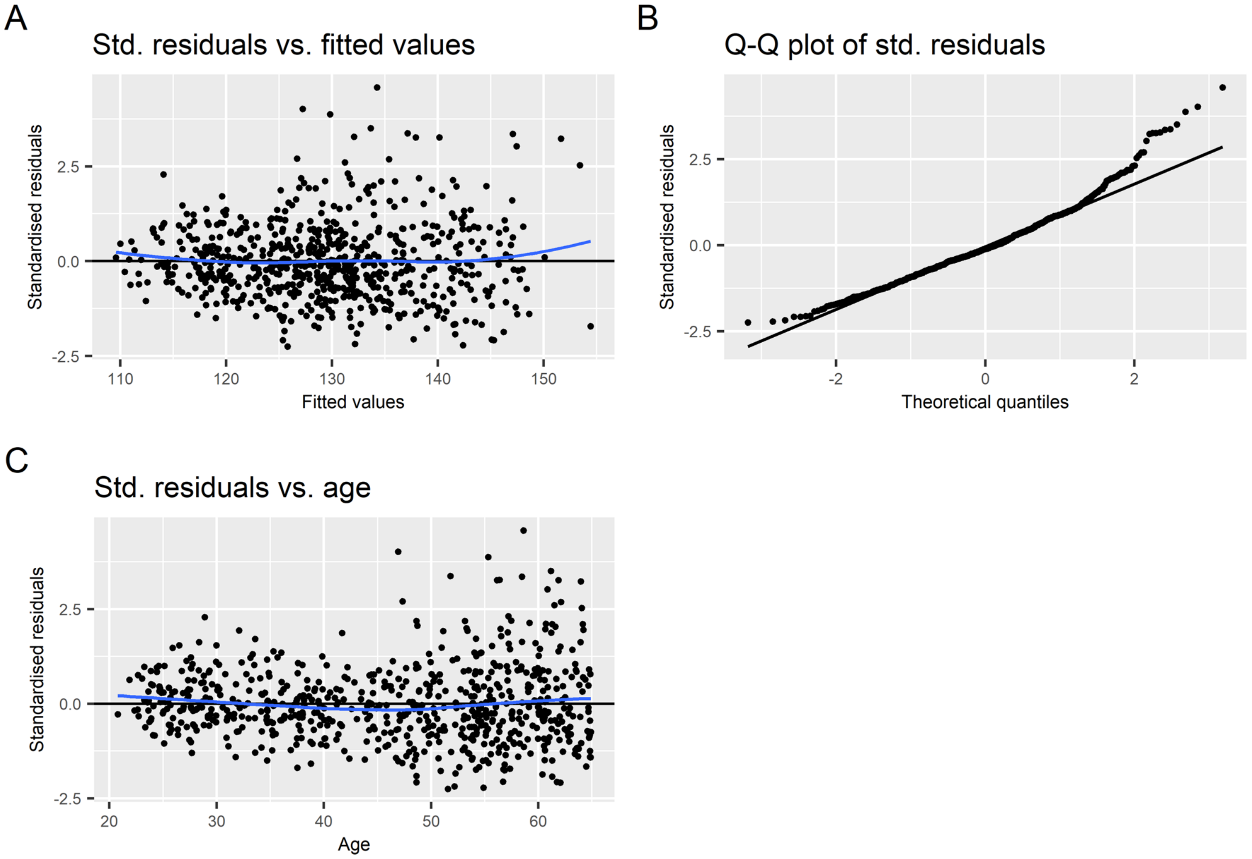

Figure 1.

5.2.3.2 Older adults

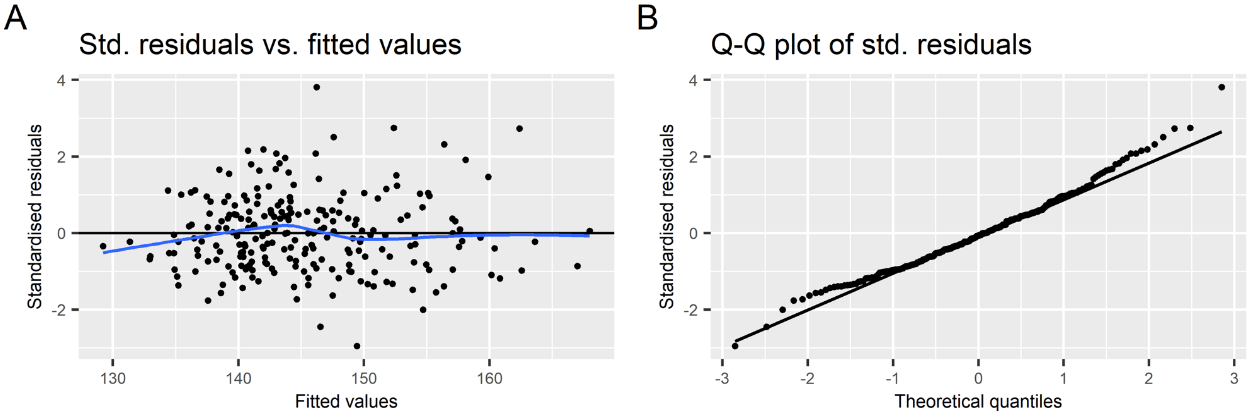

Figure 2.

5.3 Waist circumference

5.3.1 Crude model

5.3.1.1 Adults

Table A5.2.1.1. Model output from crude waist circumference-model among 773 adults

Variable	Estimate	Standard error	95% CI	
			Lower	Upper
Intercept	70.97886	2.438771	66.1914	75.76632
ilr1	8.552516	1.83354	4.953164	12.15187
ilr2	0.47755	1.766748	-2.99068	3.945785
ilr3	1.340201	1.741968	-2.07939	4.759791
ilr4	3.787097	1.551079	0.742234	6.831961
ilr5	0.25696	1.672345	-3.02596	3.539877

CI, confidence interval
ilr1-ilr5, iso-metric log-ratio coordinates representing the transformed physical behaviour composition
ilr1 represent sedentary behaviour relative to the geometric mean of the remaining behaviours in the daily physical behaviour composition

5.3.1.2 Older adults

Table A5.2.1.2. Model output from crude waist circumference-model among 280 adults

Variable	Estimate	Standard error	95% CI	
			Lower	Upper
Intercept	78.0157	3.352298	71.41617	84.61523
ilr1	10.6192	2.759159	5.187358	16.05105
ilr2	-7.5002	2.382484	-12.1905	-2.8099
ilr3	0.616125	2.36759	-4.04485	5.277104
ilr4	-0.69648	2.027211	-4.68737	3.294412
ilr5	0.352333	2.175713	-3.93091	4.635572

CI, confidence interval
ilr1-ilr5, iso-metric log-ratio coordinates representing the transformed physical behaviour composition
ilr1 represent sedentary behaviour relative to the geometric mean of the remaining behaviours in the daily physical behaviour composition

5.3.2 Adjusted model

5.3.2.1 Adults

Table A5.2.2.1. Model output from adjusted waist circumference-model among 682 adults

Variable	Estimate	Standard error	95% CI	
			Lower	Upper
Intercept	68.31307	4.828905	58.83144	77.7947
ilr1	2.185152	1.691646	-1.13642	5.506727
ilr2	0.382196	1.576828	-2.71393	3.478323
ilr3	-4.23132	1.580694	-7.33504	-1.1276
ilr4	1.973294	1.412236	-0.79966	4.746242
ilr5	-3.08485	1.542277	-6.11314	-0.05657
Age	0.200746	0.034048	0.133893	0.267599
Sex<br>Men vs. Women	8.902944	0.79553	7.340908	10.46498
Level of education	-0.825	0.264654	-1.34465	-0.30535
Smoking status<br>Previous smoker vs. Never smoker	0.458599	0.815765	-1.14317	2.060366
Smoking status<br>Smoker vs. Never smoker	-0.92343	1.11006	-3.10306	1.256187
Average units of alcohol/week	0.06578	0.044043	-0.0207	0.15226
Use of prescribed medicine<br>Yes vs. No	3.023395	1.260796	0.5478	5.498989

CI, confidence interval
ilr1-ilr5, iso-metric log-ratio coordinates representing the transformed physical behaviour composition
ilr1 represent sedentary behaviour relative to the geometric mean of the remaining behaviours in the daily physical behaviour composition

5.3.2.2 Older adults

Table A5.2.2.2. Model output from adjusted waist circumference-model among 231 adults

Variable	Estimate	Standard error	95% CI	
			Lower	Upper
Intercept	78.97129	9.487164	60.27298	97.66959
ilr1	8.611267	2.842914	3.008151	14.21438
ilr2	-4.62434	2.405714	-9.36577	0.117097
ilr3	-3.29601	2.533122	-8.28855	1.696535
ilr4	0.395618	2.108244	-3.75953	4.550768
ilr5	-1.32218	2.427567	-6.10668	3.462329
Age	-0.06823	0.125905	-0.31637	0.179921
Sex<br>Men vs. Women	8.947359	1.392517	6.202839	11.69188
Level of education	-0.47414	0.296069	-1.05767	0.10938
Smoking status<br>Previous smoker vs. Never smoker	0.353006	1.504017	-2.61127	3.317281
Smoking status<br>Smoker vs. Never smoker	0.172134	2.082937	-3.93314	4.277406
Average units of alcohol/week	0.077348	0.082247	-0.08475	0.239448
Use of prescribed medicine<br>Yes vs. No	2.89975	1.825451	-0.69804	6.497543

CI, confidence interval
ilr1-ilr5, iso-metric log-ratio coordinates representing the transformed physical behaviour composition
ilr1 represent sedentary behaviour relative to the geometric mean of the remaining behaviours in the daily physical behaviour composition

5.3.3 Model validation

5.3.3.1 Adults

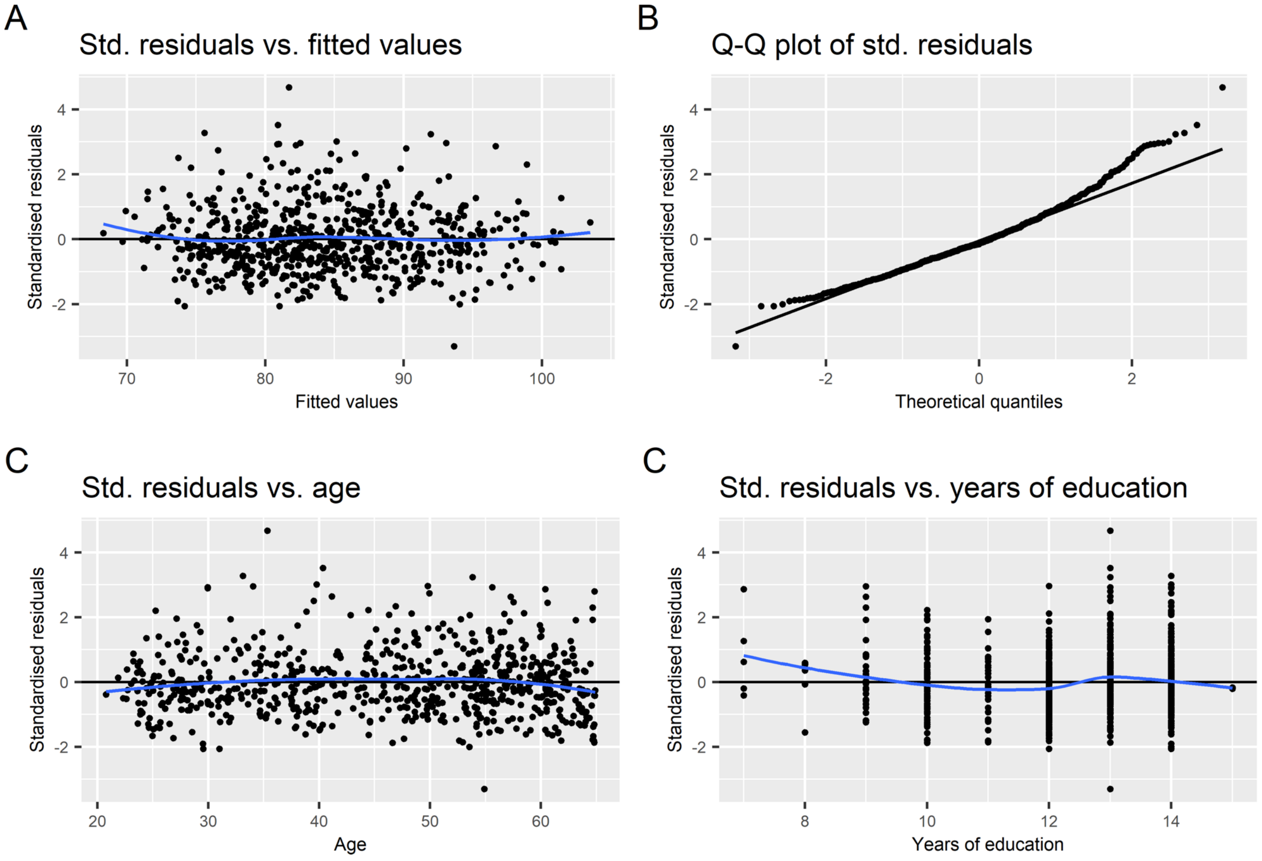

Figure 3.

5.3.3.2 Older adults

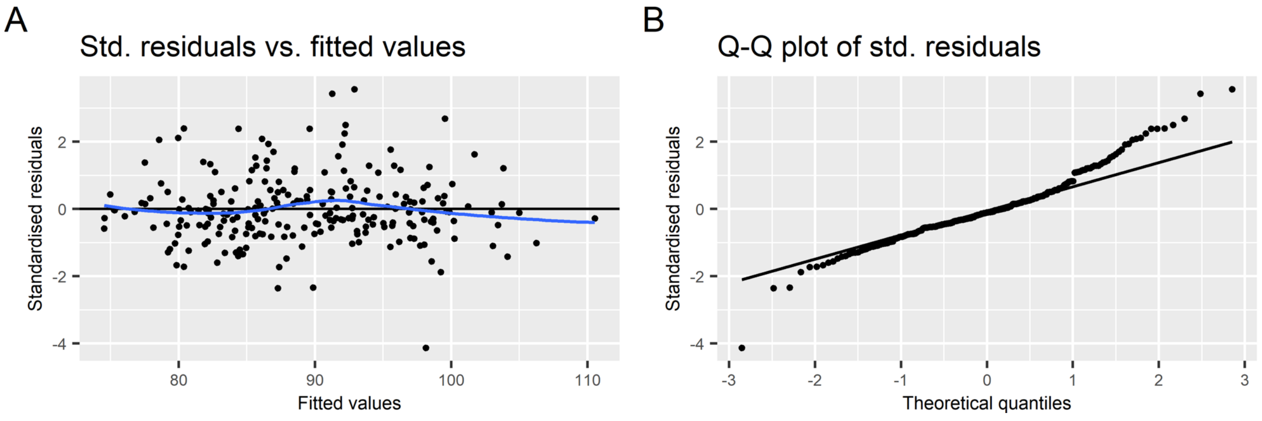

Figure 4.

5.4 Low-density lipoprotein cholesterol

5.4.1 Crude model

5.4.1.1 Adults

Table A5.3.1.1. Model output from crude low-density lipoprotein-model among 773 adults

Variable	Estimate	Standard error	95% CI	
			Lower	Upper
Intercept	2.346891	0.192929	1.968161	2.725622
ilr1	0.222387	0.145049	-0.06235	0.507128
ilr2	-0.12046	0.139766	-0.39483	0.153908
ilr3	-0.07265	0.137805	-0.34317	0.197866
ilr4	0.225648	0.122704	-0.01523	0.466524
ilr5	-0.18657	0.132297	-0.44628	0.073136

CI, confidence interval
ilr1-ilr5, iso-metric log-ratio coordinates representing the transformed physical behaviour composition
ilr1 represent sedentary behaviour relative to the geometric mean of the remaining behaviours in the daily physical behaviour composition

5.4.1.2 Older adults

Table A5.3.1.2. Model output from crude low-density lipoprotein-model among 280 adults

Variable	Estimate	Standard error	95% CI	
			Lower	Upper
Intercept	2.740967	0.258306	2.232452	3.249483
ilr1	0.012574	0.212602	-0.40597	0.431116
ilr2	0.010123	0.183578	-0.35128	0.371526
ilr3	-0.21645	0.182431	-0.5756	0.142691
ilr4	0.163368	0.156203	-0.14414	0.470879
ilr5	-0.19141	0.167646	-0.52145	0.138629

CI, confidence interval
ilr1-ilr5, iso-metric log-ratio coordinates representing the transformed physical behaviour composition
ilr1 represent sedentary behaviour relative to the geometric mean of the remaining behaviours in the daily physical behaviour composition

5.4.2 Adjusted model

5.4.2.1 Adults

Table A5.3.2.1. Model output from adjusted low-density lipoprotein-model among 682 adults

Variable	Estimate	Standard error	95% CI	
			Lower	Upper
Intercept	1.53611	0.436592	0.678854	2.393366
ilr1	0.011707	0.152946	-0.2886	0.312018
ilr2	-0.1687	0.142565	-0.44863	0.11123
ilr3	-0.39549	0.142914	-0.67611	-0.11488
ilr4	0.142686	0.127684	-0.10802	0.393395
ilr5	-0.28467	0.139441	-0.55847	-0.01088
Age	0.022461	0.003078	0.016416	0.028505
Sex<br>Men vs. Women	0.258485	0.071926	0.117257	0.399712
Level of education	-0.02168	0.023928	-0.06866	0.025303
Smoking status<br>Previous smoker vs. Never smoker	0.110571	0.073755	-0.03425	0.255391
Smoking status<br>Smoker vs. Never smoker	0.052485	0.100363	-0.14458	0.249549
Average units of alcohol/week	-0.00325	0.003982	-0.01106	0.004573
Use of prescribed medicine<br>Yes vs. No	0.091469	0.113992	-0.13236	0.315293

CI, confidence interval
ilr1-ilr5, iso-metric log-ratio coordinates representing the transformed physical behaviour composition
ilr1 represent sedentary behaviour relative to the geometric mean of the remaining behaviours in the daily physical behaviour composition

5.4.2.2 Older adults

Table A5.3.2.2. Model output from adjusted low-density lipoprotein-model among 231 adults

Variable	Estimate	Standard error	95% CI	
			Lower	Upper
Intercept	4.092403	0.824624	2.467148	5.717659
ilr1	0.034954	0.247106	-0.45207	0.521976
ilr2	-0.04828	0.209105	-0.4604	0.363849
ilr3	-0.21266	0.220179	-0.64661	0.221292
ilr4	0.102948	0.183248	-0.25822	0.464113
ilr5	-0.29359	0.211004	-0.70946	0.122282
Age	-0.02237	0.010944	-0.04394	-8.02E-04
Sex<br>Men vs. Women	-0.26924	0.121038	-0.50779	-0.03068
Level of education	0.01335	0.025734	-0.03737	0.06407
Smoking status<br>Previous smoker vs. Never smoker	-0.04127	0.130729	-0.29893	0.216381
Smoking status<br>Smoker vs. Never smoker	0.029367	0.181049	-0.32746	0.386197
Average units of alcohol/week	-0.00431	0.007149	-0.0184	0.009785
Use of prescribed medicine<br>Yes vs. No	0.251369	0.158668	-0.06135	0.564089

CI, confidence interval
ilr1-ilr5, iso-metric log-ratio coordinates representing the transformed physical behaviour composition
ilr1 represent sedentary behaviour relative to the geometric mean of the remaining behaviours in the daily physical behaviour composition

5.4.3 Model validation

5.4.3.1 Adults

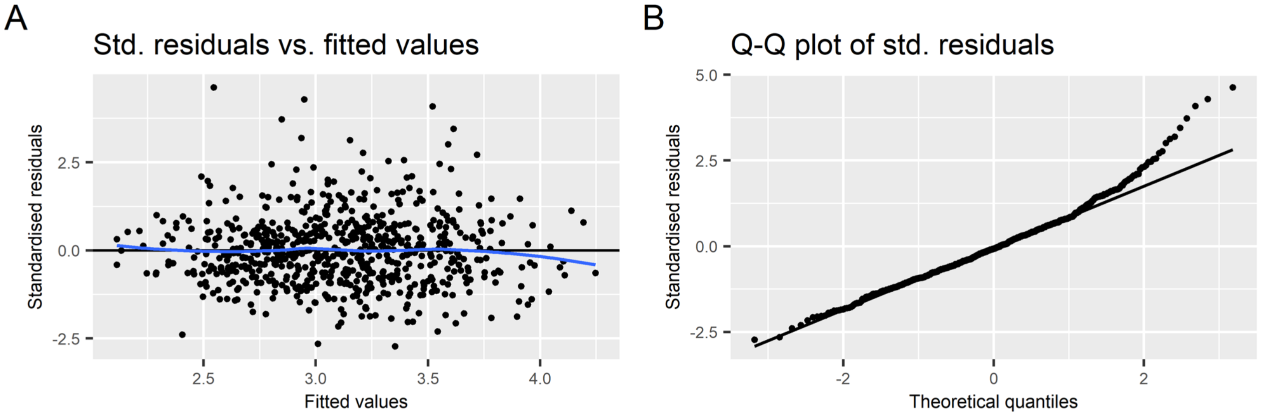

Figure 5.

5.4.3.2 Older adults

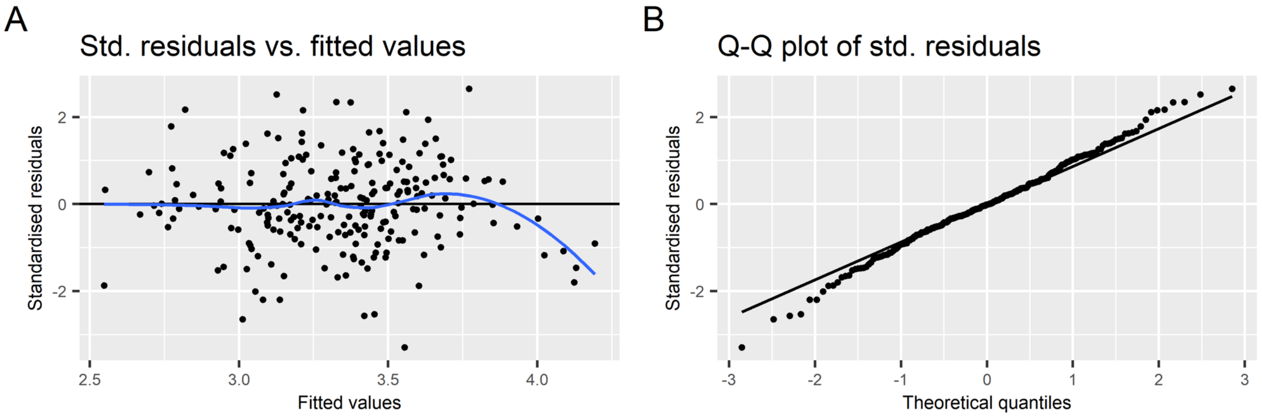

Figure 6.

6 Time reallocations

6.1 Systolic blood pressure

6.1.1 Crude

Table A6.1.1. Estimated crude differences in systolic blood pressure given time reallocations between sedentary behaviour and walking and sedentary behaviour and high intensity physical activity among 773 adults and 280 older adults in the fifth examination of the Copenhagen City Heart Study (Denmark)

Reallocation (min)	**Adults** *n* = 773 Estimated difference in mm Hg w. 95% CI			**Older adults** *n* = 280 Estimated difference in mm Hg w. 95% CI		
Sedentary behaviour – walking	**Estimate**	**Lower**	**Upper**	**Estimate**	**Lower**	**Upper**
-60 (sedentary behaviour → walking)	-0.610	-2.857	1.638	**-5.473**	**-9.317**	**-1.629**
-50	-0.490	-2.429	1.450	**-4.715**	**-8.040**	**-1.390**
-40	-0.376	-1.987	1.235	**-3.911**	**-6.680**	**-1.141**
-30	-0.269	-1.527	0.989	**-3.051**	**-5.222**	**-0.880**
-20	-0.170	-1.047	0.706	**-2.124**	**-3.643**	**-0.605**
-10	-0.080	-0.540	0.379	**-1.114**	**-1.915**	**-0.314**
0 (reference composition)	0.000	0.000	0.000	0.000	0.000	0.000
10	0.069	-0.445	0.582	**1.250**	**0.342**	**2.158**
20	0.124	-0.973	1.220	**2.681**	**0.721**	**4.642**
30	0.161	-1.613	1.934	**4.370**	**1.153**	**7.586**
40	0.174	-2.407	2.754	**6.446**	**1.665**	**11.226**
50	0.153	-3.432	3.737	**9.177**	**2.312**	**16.042**
60 (walking → sedentary behaviour)	0.075	-4.843	4.994	**13.256**	**3.235**	**23.277**
Sedentary behaviour – HIPA						
-12 (sedentary behaviour → HIPA)	**-1.797**	**-2.540**	**-1.054**	-	-	-
-10	**-1.557**	**-2.204**	**-0.910**	-	-	-
-8	**-1.299**	**-1.841**	**-0.757**	-	-	-
-6	**-1.020**	**-1.447**	**-0.592**	**-1.983**	**-3.346**	**-0.620**
-4	**-0.715**	**-1.016**	**-0.414**	**-1.443**	**-2.438**	**-0.447**
-2	**-0.378**	**-0.537**	**-0.218**	**-0.799**	**-1.353**	**-0.246**
0 (reference composition)	0.000	0.000	0.000	0.000	0.000	0.000
2	**0.431**	**0.246**	**0.615**	**1.062**	**0.319**	**1.805**
4	**0.933**	**0.532**	**1.335**	**2.667**	**0.791**	**4.543**
6	**1.540**	**0.873**	**2.206**	**6.153**	**1.789**	**10.517**
8	**2.309**	**1.302**	**3.316**	-	-	-
10	**3.371**	**1.889**	**4.854**	-	-	-
12 (HIPA → sedentary behaviour)	**5.126**	**2.848**	**7.403**	-	-	-

n, number of observations included in model
mm Hg, mm of mercury
CI, confidence interval
Reference composition corresponds to A) 579.8 min sedentary behaviour, 193.4 min standing, 71.1 min moving, 85.5 min walking, 14.2 min HIPA, and 496.1 min in bed among adults, and B) 589.1 min sedentary behaviour, 186.5 min standing, 72.9 min moving, 74.9 min walking, 6.9 min HIPA, and 509.7 min in bed among older adults (i.e., geometric mean).
HIPA, high-intensity physical activity which consists of climbing stairs (up/down), running, cycling and rowing

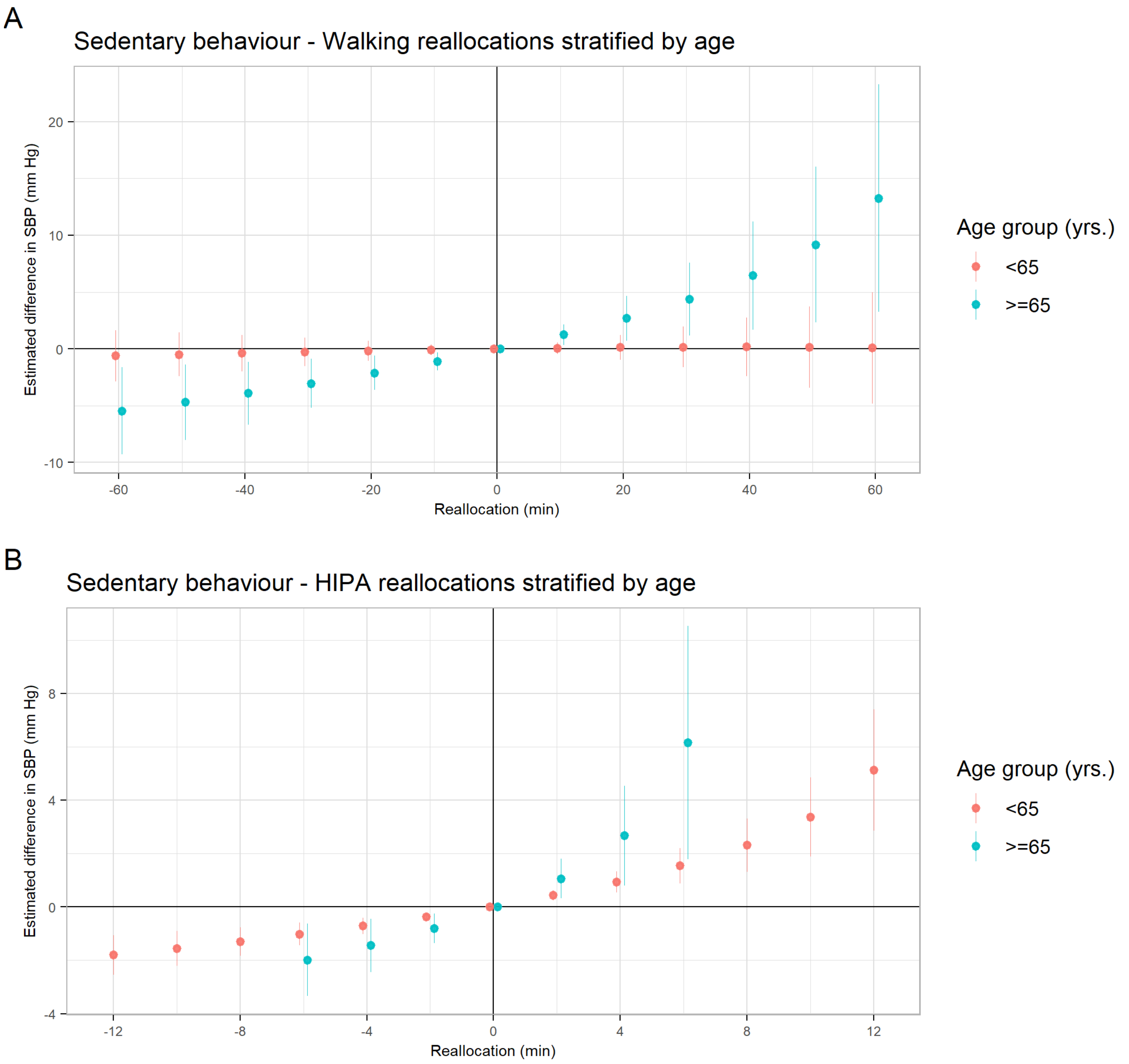

Figure 7. Illustration of estimated crude differences in systolic blood pressure (mm Hg) given the reallocation of time between **A)** sedentary behaviour and walking, and **B)** sedentary behaviour and high intensity physical activity (HIPA) among 773 adults and 280 older adults. A negative reallocated value reflect the pairwise reallocation of time from sedentary behaviour to walking or HIPA, while a positive value reflect the increase in sedentary behaviour at the cost of walking or HIPA. The origin represent the group-specific geometric mean composition. Vertical lines corresponds to the 95% confidence intervals. HIPA is high-intensity physical activity (i.e., sum of climbing stairs, running, cycling and rowing).

6.1.2 Adjusted

Table A6.1.2. Estimated adjusted differences in systolic blood pressure given time reallocations between sedentary behaviour and walking and sedentary behaviour and high intensity physical activity among 773 adults and 280 older adults in the fifth examination of the Copenhagen City Heart Study (Denmark)

**Reallocation (min)**	**Adults *n* = 682 Estimated difference in mm Hg w. 95% CI**			**Older adults *n* = 231 Estimated difference in mm Hg w. 95% CI**		
Sedentary behaviour – walking	**Estimate**	**Lower**	**Upper**	**Estimate**	**Lower**	**Upper**
-60 (sedentary behaviour → walking)	0.820	-1.261	2.901	-3.509	-7.956	0.939
-50	0.729	-1.067	2.524	-3.006	-6.852	0.839
-40	0.623	-0.869	2.114	-2.480	-5.682	0.722
-30	0.500	-0.665	1.665	-1.924	-4.432	0.584
-20	0.358	-0.453	1.169	-1.332	-3.085	0.422
-10	0.193	-0.233	0.618	-0.694	-1.618	0.230
0 (reference composition)	0.000	0.000	0.000	0.000	0.000	0.000
10	-0.227	-0.702	0.248	0.769	-0.278	1.816
20	-0.498	-1.513	0.517	1.638	-0.620	3.896
30	-0.826	-2.468	0.815	2.650	-1.052	6.352
40	-1.235	-3.624	1.153	3.877	-1.620	9.375
50	-1.764	-5.083	1.554	5.470	-2.418	13.358
60 (walking → sedentary behaviour)	-2.493	-7.046	2.061	7.815	-3.687	19.317
Sedentary behaviour – HIPA						
-12 (sedentary behaviour → HIPA)	-0.685	-1.432	0.062	-	-	-
-10	-0.593	-1.242	0.057	-	-	-
-8	-0.494	-1.038	0.051	-	-	-
-6	-0.387	-0.817	0.042	-1.062	-2.612	0.487
-4	-0.271	-0.573	0.031	-0.770	-1.902	0.362
-2	-0.143	-0.303	0.017	-0.425	-1.054	0.205
0 (reference composition)	0.000	0.000	0.000	0.000	0.000	0.000
2	0.163	-0.022	0.347	0.559	-0.285	1.404
4	0.352	-0.051	0.755	1.397	-0.736	3.530
6	0.579	-0.089	1.248	3.197	-1.764	8.158
8	0.867	-0.142	1.877	-	-	-
10	1.264	-0.221	2.749	-	-	-
12 (HIPA → sedentary behaviour)	1.916	-0.364	4.197	-	-	-

n, number of observations included in adjusted model
mm Hg, mm of mercury
CI, confidence interval
Reference composition corresponds to A) 579.8 min sedentary behaviour, 193.4 min standing, 71.1 min moving, 85.5 min walking, 14.2 min HIPA, and 496.1 min in bed among adults, and B) 589.1 min sedentary behaviour, 186.5 min standing, 72.9 min moving, 74.9 min walking, 6.9 min HIPA, and 509.7 min in bed among older adults (i.e., geometric mean).
HIPA, high-intensity physical activity which consists of climbing stairs (up/down), running, cycling and rowing

6.2 Waist circumference

6.2.1 Crude

Table A6.2.1. Estimated crude differences in waist circumference given time reallocations between sedentary behaviour and walking and sedentary behaviour and high intensity physical activity among 773 adults and 280 older adults in the fifth examination of the Copenhagen City Heart Study (Denmark)

Reallocation (min)	Adults *n* = 773 Estimated difference in cm w. 95% CI			Older adults *n* = 280 Estimated difference in cm w. 95% CI		
Sedentary behaviour – walking	**Estimate**	**Lower**	**Upper**	**Estimate**	**Lower**	**Upper**
-60 (sedentary behaviour → walking)	-0.301	-1.842	1.239	-1.635	-3.724	0.455
-50	-0.226	-1.555	1.103	-1.375	-3.183	0.432
-40	-0.160	-1.264	0.944	-1.113	-2.619	0.393
-30	-0.103	-0.965	0.759	-0.846	-2.026	0.334
-20	-0.056	-0.656	0.544	-0.573	-1.399	0.252
-10	-0.021	-0.336	0.294	-0.292	-0.728	0.143
0 (reference composition)	0.000	0.000	0.000	0.000	0.000	0.000
10	0.004	-0.347	0.356	0.308	-0.186	0.801
20	-0.012	-0.763	0.740	0.637	-0.429	1.703
30	-0.054	-1.269	1.161	0.997	-0.751	2.746
40	-0.134	-1.902	1.635	1.407	-1.192	4.006
50	-0.266	-2.722	2.190	1.900	-1.832	5.632
60 (walking → sedentary behaviour)	-0.486	-3.857	2.884	2.568	-2.879	8.016
Sedentary behaviour – HIPA						
-12 (sedentary behaviour → HIPA)	**-2.263**	**-2.772**	**-1.753**	-	-	-
-10	**-1.963**	**-2.406**	**-1.520**	-	-	-
-8	**-1.640**	**-2.012**	**-1.269**	-	-	-
-6	**-1.289**	**-1.582**	**-0.996**	-0.041	-0.782	0.700
-4	**-0.905**	**-1.111**	**-0.699**	-0.023	-0.564	0.518
-2	**-0.479**	**-0.588**	**-0.369**	-0.009	-0.310	0.292
0 (reference composition)	0.000	0.000	0.000	0.000	0.000	0.000
2	**0.548**	**0.421**	**0.674**	0.001	-0.403	0.405
4	**1.189**	**0.914**	**1.464**	-0.015	-1.035	1.005
6	**1.966**	**1.509**	**2.423**	-0.089	-2.462	2.283
8	**2.954**	**2.264**	**3.644**	-	-	-
10	**4.324**	**3.308**	**5.340**	-	-	-
12 (HIPA → sedentary behaviour)	**6.593**	**5.033**	**8.154**	-	-	-

n, number of observations included in model
mm Hg, mm of mercury
CI, confidence interval
Reference composition corresponds to A) 579.8 min sedentary behaviour, 193.4 min standing, 71.1 min moving, 85.5 min walking, 14.2 min HIPA, and 496.1 min in bed among adults, and B) 589.1 min sedentary behaviour, 186.5 min standing, 72.9 min moving, 74.9 min walking, 6.9 min HIPA, and 509.7 min in bed among older adults (i.e., geometric mean).
HIPA, high-intensity physical activity which consists of climbing stairs (up/down), running, cycling and rowing

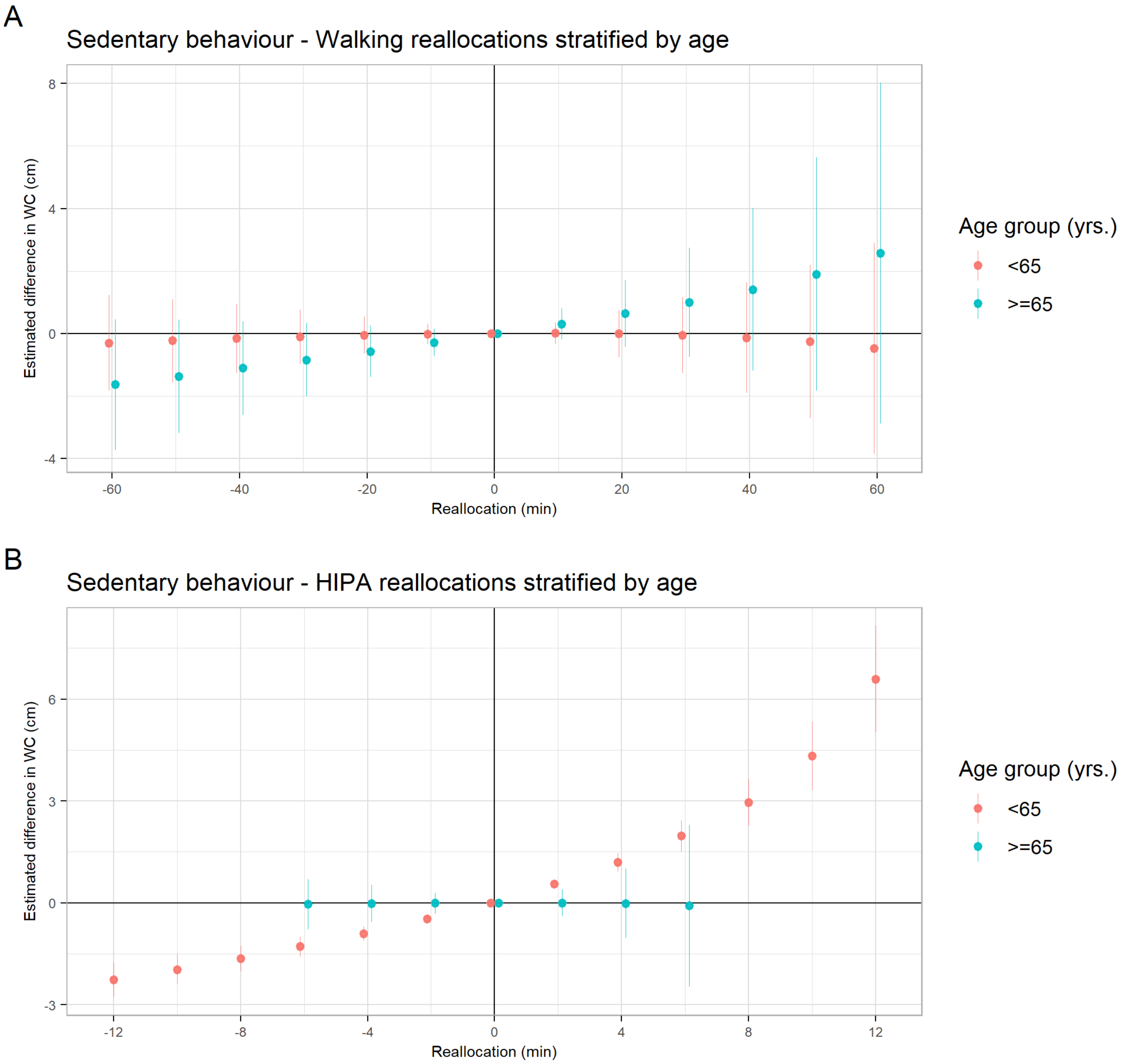

Figure 8. Illustration of estimated crude differences in waist circumference (cm) given the reallocation of time between **A)** sedentary behaviour and walking, and **B)** sedentary behaviour and high intensity physical activity (HIPA) among 773 adults and 280 older adults. A negative reallocated value reflect the pairwise reallocation of time from sedentary behaviour to walking or HIPA, while a positive value reflect the increase in sedentary behaviour at the cost of walking or HIPA. The origin represent the group-specific geometric mean composition. Vertical lines corresponds to the 95% confidence intervals. HIPA is high-intensity physical activity (i.e., sum of climbing stairs, running, cycling and rowing).

6.2.2 Adjusted

Table A6.2.2. Estimated adjusted differences in waist circumference given time reallocations between sedentary behaviour and walking and sedentary behaviour and high intensity physical activity among 773 adults and 280 older adults in the fifth examination of the Copenhagen City Heart Study (Denmark)

**Reallocation (min)**	**Adults** *n* **= 682** **Estimated difference in cm w. 95% CI**			**Older adults** *n* **= 231** **Estimated difference in cm w. 95% CI**		
Sedentary behaviour – walking	**Estimate**	**Lower**	**Upper**	**Estimate**	**Lower**	**Upper**
-60 (sedentary behaviour → walking)	1.031	-0.315	2.376	-0.411	-2.626	1.803
-50	0.902	-0.259	2.062	-0.321	-2.235	1.594
-40	0.759	-0.205	1.723	-0.238	-1.832	1.356
-30	0.600	-0.152	1.353	-0.163	-1.412	1.086
-20	0.424	-0.101	0.948	-0.097	-0.970	0.776
-10	0.225	-0.050	0.500	-0.042	-0.502	0.418
0 (reference composition)	0.000	0.000	0.000	0.000	0.000	0.000
10	-0.258	-0.565	0.049	0.027	-0.494	0.548
20	-0.558	-1.214	0.098	0.034	-1.091	1.158
30	-0.914	-1.975	0.146	0.014	-1.830	1.857
40	-1.349	-2.893	0.195	-0.046	-2.783	2.691
50	-1.900	-4.045	0.245	-0.171	-4.098	3.757
60 (walking → sedentary behaviour)	-2.646	-5.589	0.298	-0.427	-6.154	5.300
Sedentary behaviour – HIPA						
-12 (sedentary behaviour → HIPA)	**-1.423**	**-1.906**	**-0.940**	-	-	-
-10	**-1.237**	**-1.657**	**-0.817**	-	-	-
-8	**-1.036**	**-1.387**	**-0.684**	-	-	-
-6	**-0.816**	**-1.093**	**-0.538**	-0.506	-1.277	0.266
-4	**-0.574**	**-0.769**	**-0.378**	-0.364	-0.928	0.199
-2	**-0.304**	**-0.408**	**-0.201**	-0.200	-0.513	0.114
0 (reference composition)	0.000	0.000	0.000	0.000	0.000	0.000
2	**0.350**	**0.230**	**0.469**	0.259	-0.162	0.679
4	**0.761**	**0.500**	**1.021**	0.639	-0.423	1.702
6	**1.261**	**0.829**	**1.693**	1.443	-1.027	3.913
8	**1.901**	**1.248**	**2.553**	-	-	-
10	**2.791**	**1.831**	**3.751**	-	-	-
12 (HIPA → sedentary behaviour)	**4.274**	**2.800**	**5.748**	-	-	-

n, number of observations included in adjusted model
mm Hg, mm of mercury
CI, confidence interval
Reference composition corresponds to A) 579.8 min sedentary behaviour, 193.4 min standing, 71.1 min moving, 85.5 min walking, 14.2 min HIPA, and 496.1 min in bed among adults, and B) 589.1 min sedentary behaviour, 186.5 min standing, 72.9 min moving, 74.9 min walking, 6.9 min HIPA, and 509.7 min in bed among older adults (i.e., geometric mean).
HIPA, high-intensity physical activity which consists of climbing stairs (up/down), running, cycling and rowing

6.3 Low-density lipoprotein cholesterol

6.3.1 Crude

Table A6.3.1. Estimated crude differences in low-density lipoprotein cholesterol given time reallocations between sedentary behaviour and walking and sedentary behaviour and high intensity physical activity among 773 adults and 280 older adults in the fifth examination of the Copenhagen City Heart Study (Denmark)

**Reallocation (min)**	**Adults *n* = 773 Estimated difference in mmol/L w. 95% CI**			**Older adults *n* = 280 Estimated difference in mmol/L w. 95% CI**		
Sedentary behaviour – walking	**Estimate**	**Lower**	**Upper**	**Estimate**	**Lower**	**Upper**
-60 (sedentary behaviour → walking)	0.080	-0.042	0.202	0.111	-0.050	0.272
-50	0.070	-0.035	0.175	0.097	-0.042	0.236
-40	0.059	-0.028	0.146	0.081	-0.035	0.197
-30	0.047	-0.021	0.115	0.064	-0.027	0.155
-20	0.033	-0.014	0.081	0.045	-0.019	0.108
-10	0.018	-0.007	0.043	0.024	-0.010	0.057
0 (reference composition)	0.000	0.000	0.000	0.000	0.000	0.000
10	-0.020	-0.048	0.007	-0.027	-0.065	0.011
20	-0.044	-0.104	0.015	-0.059	-0.141	0.023
30	-0.073	-0.169	0.024	-0.097	-0.232	0.037
40	-0.107	-0.247	0.033	-0.145	-0.346	0.055
50	-0.152	-0.346	0.043	-0.210	-0.497	0.078
60 (walking → sedentary behaviour)	-0.212	-0.478	0.055	-0.308	-0.728	0.112
Sedentary behaviour – HIPA						
-12 (sedentary behaviour → HIPA)	**-0.137**	**-0.178**	**-0.097**	-	-	-
-10	**-0.119**	**-0.154**	**-0.084**	-	-	-
-8	**-0.100**	**-0.129**	**-0.071**	-	-	-
-6	**-0.079**	**-0.102**	**-0.056**	**-0.090**	**-0.147**	**-0.033**
-4	**-0.055**	**-0.072**	**-0.039**	**-0.066**	**-0.107**	**-0.024**
-2	**-0.029**	**-0.038**	**-0.021**	**-0.037**	**-0.060**	**-0.013**
0 (reference composition)	0.000	0.000	0.000	0.000	0.000	0.000
2	**0.034**	**0.024**	**0.044**	**0.049**	**0.018**	**0.080**
4	**0.073**	**0.052**	**0.095**	**0.124**	**0.045**	**0.202**
6	**0.122**	**0.085**	**0.158**	**0.288**	**0.105**	**0.471**
8	**0.183**	**0.129**	**0.238**	-	-	-
10	**0.269**	**0.189**	**0.349**	-	-	-
12 (HIPA → sedentary behaviour)	**0.412**	**0.288**	**0.535**	-	-	-

n, number of observations included in model
mm Hg, mm of mercury
CI, confidence interval
Reference composition corresponds to A) 579.8 min sedentary behaviour, 193.4 min standing, 71.1 min moving, 85.5 min walking, 14.2 min HIPA, and 496.1 min in bed among adults, and B) 589.1 min sedentary behaviour, 186.5 min standing, 72.9 min moving, 74.9 min walking, 6.9 min HIPA, and 509.7 min in bed among older adults (i.e., geometric mean).
HIPA, high-intensity physical activity which consists of climbing stairs (up/down), running, cycling and rowing

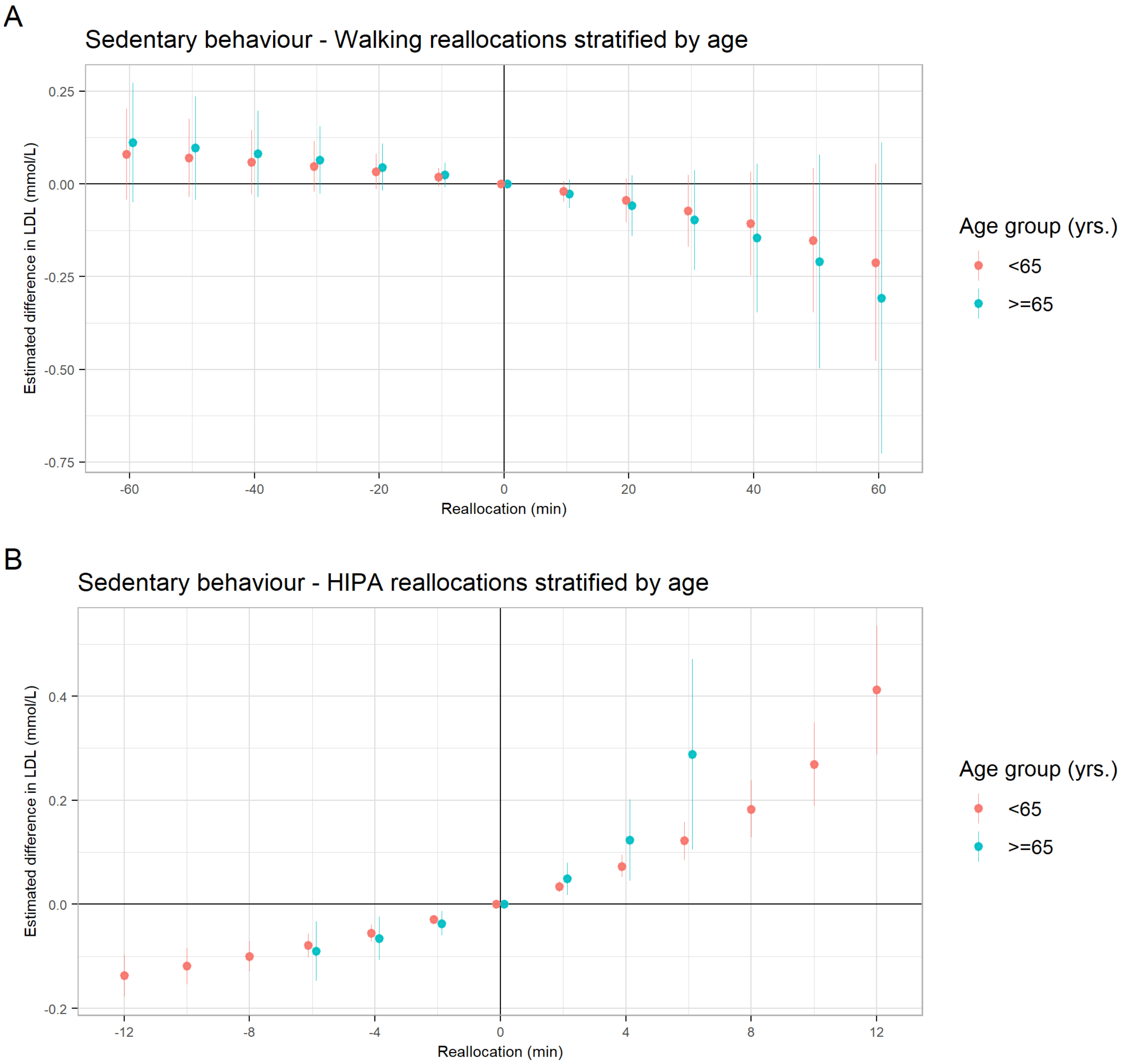

Figure 9. Illustration of estimated crude differences in low-density lipoprotein cholesterol (mmol/L) given the reallocation of time between **A)** sedentary behaviour and walking, and **B)** sedentary behaviour and high intensity physical activity (HIPA) among 773 adults and 280 older adults. A negative reallocated value reflect the pairwise reallocation of time from sedentary behaviour to walking or HIPA, while a positive value reflect the increase in sedentary behaviour at the cost of walking or HIPA. The origin represent the group-specific geometric mean composition. Vertical lines corresponds to the 95% confidence intervals. HIPA is high-intensity physical activity (i.e., sum of climbing stairs, running, cycling and rowing).

6.3.2 Adjusted

Table A6.3.2. Estimated adjusted differences in low-density lipoprotein cholesterol given time reallocations between sedentary behaviour and walking and sedentary behaviour and high intensity physical activity among 773 adults and 280 older adults in the fifth examination of the Copenhagen City Heart Study (Denmark)

**Reallocation (min)**	**Adults** *n* **= 682 Estimated difference in mmol/L w. 95% CI**			**Older adults** *n* **= 231 Estimated difference in mmol/L w. 95% CI**		
Sedentary behaviour – walking	**Estimate**	**Lower**	**Upper**	**Estimate**	**Lower**	**Upper**
-60 (sedentary behaviour → walking)	**0.140**	**0.019**	**0.262**	0.085	-0.108	0.277
-50	**0.122**	**0.017**	**0.227**	0.074	-0.093	0.240
-40	**0.101**	**0.014**	**0.189**	0.062	-0.077	0.200
-30	**0.080**	**0.011**	**0.148**	0.049	-0.060	0.157
-20	**0.056**	**0.008**	**0.103**	0.034	-0.042	0.110
-10	**0.029**	**0.004**	**0.054**	0.018	-0.022	0.058
0 (reference composition)	0.000	0.000	0.000	0.000	0.000	0.000
10	**-0.033**	**-0.061**	**-0.005**	-0.021	-0.066	0.024
20	**-0.071**	**-0.130**	**-0.011**	-0.045	-0.143	0.052
30	**-0.115**	**-0.210**	**-0.019**	-0.075	-0.235	0.085
40	**-0.167**	**-0.307**	**-0.028**	-0.112	-0.350	0.126
50	**-0.233**	**-0.427**	**-0.039**	-0.162	-0.504	0.179
60 (walking → sedentary behaviour)	**-0.321**	**-0.587**	**-0.055**	-0.239	-0.737	0.259
Sedentary behaviour – HIPA						
-12 (sedentary behaviour → HIPA)	**-0.068**	**-0.111**	**-0.024**	-	-	-
-10	**-0.059**	**-0.097**	**-0.021**	-	-	-
-8	**-0.049**	**-0.081**	**-0.018**	-	-	-
-6	**-0.039**	**-0.064**	**-0.014**	**-0.115**	**-0.182**	**-0.048**
-4	**-0.027**	**-0.045**	**-0.010**	**-0.084**	**-0.133**	**-0.035**
-2	**-0.015**	**-0.024**	**-0.005**	**-0.047**	**-0.074**	**-0.019**
0 (reference composition)	0.000	0.000	0.000	0.000	0.000	0.000
2	**0.017**	**0.006**	**0.028**	**0.063**	**0.026**	**0.099**
4	**0.037**	**0.013**	**0.060**	**0.158**	**0.066**	**0.250**
6	**0.061**	**0.022**	**0.100**	**0.367**	**0.153**	**0.582**
8	**0.092**	**0.033**	**0.151**	-	-	-
10	**0.135**	**0.048**	**0.222**	-	-	-
12 (HIPA → sedentary behaviour)	**0.207**	**0.074**	**0.340**	-	-	-

n, number of observations included in adjusted model
mm Hg, mm of mercury
CI, confidence interval
Reference composition corresponds to A) 579.8 min sedentary behaviour, 193.4 min standing, 71.1 min moving, 85.5 min walking, 14.2 min HIPA, and 496.1 min in bed among adults, and B) 589.1 min sedentary behaviour, 186.5 min standing, 72.9 min moving, 74.9 min walking, 6.9 min HIPA, and 509.7 min in bed among older adults (i.e., geometric mean).
HIPA, high-intensity physical activity which consists of climbing stairs (up/down), running, cycling and rowing
